# RNA-seq driven expression and enrichment analysis to investigate CVD genes with associated phenotypes among high-risk heart failure patients

**DOI:** 10.1186/s40246-021-00367-8

**Published:** 2021-11-13

**Authors:** Zeeshan Ahmed, Saman Zeeshan, Bruce T. Liang

**Affiliations:** 1 Rutgers Institute for Health, Health Care Policy and Aging Research, Rutgers University, 112 Paterson Street, New Brunswick, NJ 08901 USA; 2 Department of Medicine, Robert Wood Johnson Medical School, Rutgers Biomedical and Health Sciences, 125 Paterson St, New Brunswick, NJ USA; 3 Rutgers Cancer Institute of New Jersey, Rutgers University, 195 Little Albany St, New Brunswick, NJ USA; 4 Department of Genetics and Genome Sciences, UConn Health, 400 Farmington Ave, Farmington, CT USA; 5 Pat and Jim Calhoun Cardiology Center, UConn School of Medicine, University of Connecticut Health Center, 263 Farmington Ave, Farmington, CT USA

**Keywords:** Cardiovascular, Disease, Expression, Enrichment, Gene, Heart failure, RNA-seq

## Abstract

**Background:**

Heart failure (HF) is one of the most common complications of cardiovascular diseases (CVDs) and among the leading causes of death in the US. Many other CVDs can lead to increased mortality as well. Investigating the genetic epidemiology and susceptibility to CVDs is a central focus of cardiology and biomedical life sciences. Several studies have explored expression of key CVD genes specially in HF, yet new targets and biomarkers for early diagnosis are still missing to support personalized treatment. Lack of gender-specific cardiac biomarker thresholds in men and women may be the reason for CVD underdiagnosis in women, and potentially increased morbidity and mortality as a result, or conversely, an overdiagnosis in men. In this context, it is important to analyze the expression and enrichment of genes with associated phenotypes and disease-causing variants among high-risk CVD populations.

**Methods:**

We performed RNA sequencing focusing on key CVD genes with a great number of genetic associations to HF. Peripheral blood samples were collected from a broad age range of adult male and female CVD patients. These patients were clinically diagnosed with CVDs and CMS/HCC HF, as well as including cardiomyopathy, hypertension, obesity, diabetes, asthma, high cholesterol, hernia, chronic kidney, joint pain, dizziness and giddiness, osteopenia of multiple sites, chest pain, osteoarthritis, and other diseases.

**Results:**

We report RNA-seq driven case–control study to analyze patterns of expression in genes and differentiating the pathways, which differ between healthy and diseased patients. Our in-depth gene expression and enrichment analysis of RNA-seq data from patients with mostly HF and other CVDs on differentially expressed genes and CVD annotated genes revealed 4,885 differentially expressed genes (DEGs) and regulation of 41 genes known for HF and 23 genes related to other CVDs, with 15 DEGs as significantly expressed including four genes already known (FLNA, CST3, LGALS3, and HBA1) for HF and CVDs with the enrichment of many pathways. Furthermore, gender and ethnic group specific analysis showed shared and unique genes between the genders, and among different races. Broadening the scope of the results in clinical settings, we have linked the CVD genes with ICD codes.

**Conclusions:**

Many pathways were found to be enriched, and gender-specific analysis showed shared and unique genes between the genders. Additional testing of these genes may lead to the development of new clinical tools to improve diagnosis and prognosis of CVD patients.

**Supplementary Information:**

The online version contains supplementary material available at 10.1186/s40246-021-00367-8.

## Introduction

Cardiovascular diseases (CVDs) are among the leading causes of morbidity and mortality in the US [1–3]. Among all CVDs, ischemic and nonischemic heart failure (HF) and stroke are the most common causes of death [4, 5]. According to the Centers for Disease Control and Prevention (CDC), a person with a CVD dies every 36 s in the US, totaling 655,000 deaths each year [6]. Numerous studies have reported that age and gender are the socio-demographic characteristics most frequently associated with CVDs [7–9], yet the molecular underpinnings of these findings are not yet clear.

Establishing a deeper understanding of CVDs by investigating human genetic epidemiology and susceptibility to CVDs is a central focus of cardiology and biomedical life sciences today [10]. Our evolving understanding of CVD has led to the realization that to effectively diagnose and treat CVD patients, a precision medicine approach is essential [11]. To identify patients during the preclinical stages of CVD and provide the most efficacious personalized treatment, it is essential to analyze the expression of human genes with disease-causing variants, along with associated phenotypes among high-risk CVD populations, mainly those with hypertension, obesity, type 2 diabetes mellitus, asthma, high cholesterol, hernia, chronic kidney, joint pain, myalgia, dizziness and giddiness, osteopenia of multiple sites, chest pain, osteoarthritis, and related diseases [12]. The apparent challenge here is to identify and quantify the genes that contribute to major CVD etiologies specifically HF [13].

Heart diseases like HF happens gradually over time when the muscles of the heart become weak and have difficulty pumping enough blood to nourish your body's many cells. HF and most other CVD clinical phenotypes exist due to complicated relations between genetic and ecological factors [14]. Several recently published studies have shown that gene expression analysis is a proven method for understanding and discovering novel and sensitive biomarkers of CVDs [15]. Gene expression and classification analysis have shown strong correlations of age and gender with obstructive coronary arterial disease (CAD) [16], differentiated ischemic and non-ischemic cardiomyopathy conditions [17], identified genes related to HF [18], and discovered differentially regulated genes linked with recurrent cardiovascular outcomes in first-time acute myocardial infarction (AMI) patients [19]. The susceptibility to heart failure depends on complex and heterogeneous genetic predisposition [20]. This genetic and therefore heritable component has been determined in many HF studies [21–24]. These studies clearly demonstrated the presence of genetic factors as determinants of heart failure. They also showed the relevance of genetic factors as independent risk factors for heart failure.

In this study, we investigated genes responsible for pathophysiological processes in CVDs with a focus on HF. In addition, our expression profiling revealed new gene-disease associations that may lead to the development of new clinical tools to improve diagnosis and prognosis of patients. RNA sequencing (RNA-seq) analyses are used to quantify expressed genes [25]. We performed an RNA-seq analysis from peripheral blood of diverse CVD patients and focusing on HF and other CVD genes. We used gene expression analysis to identify changes in mRNA abundance [26] that correlate with CVDs to precisely stratify, classify, and distinguish gender- and age-based patient populations to CVD risks and subtypes by using genomic phenotypes [27].

## Material and methods

**Fig. 1:**
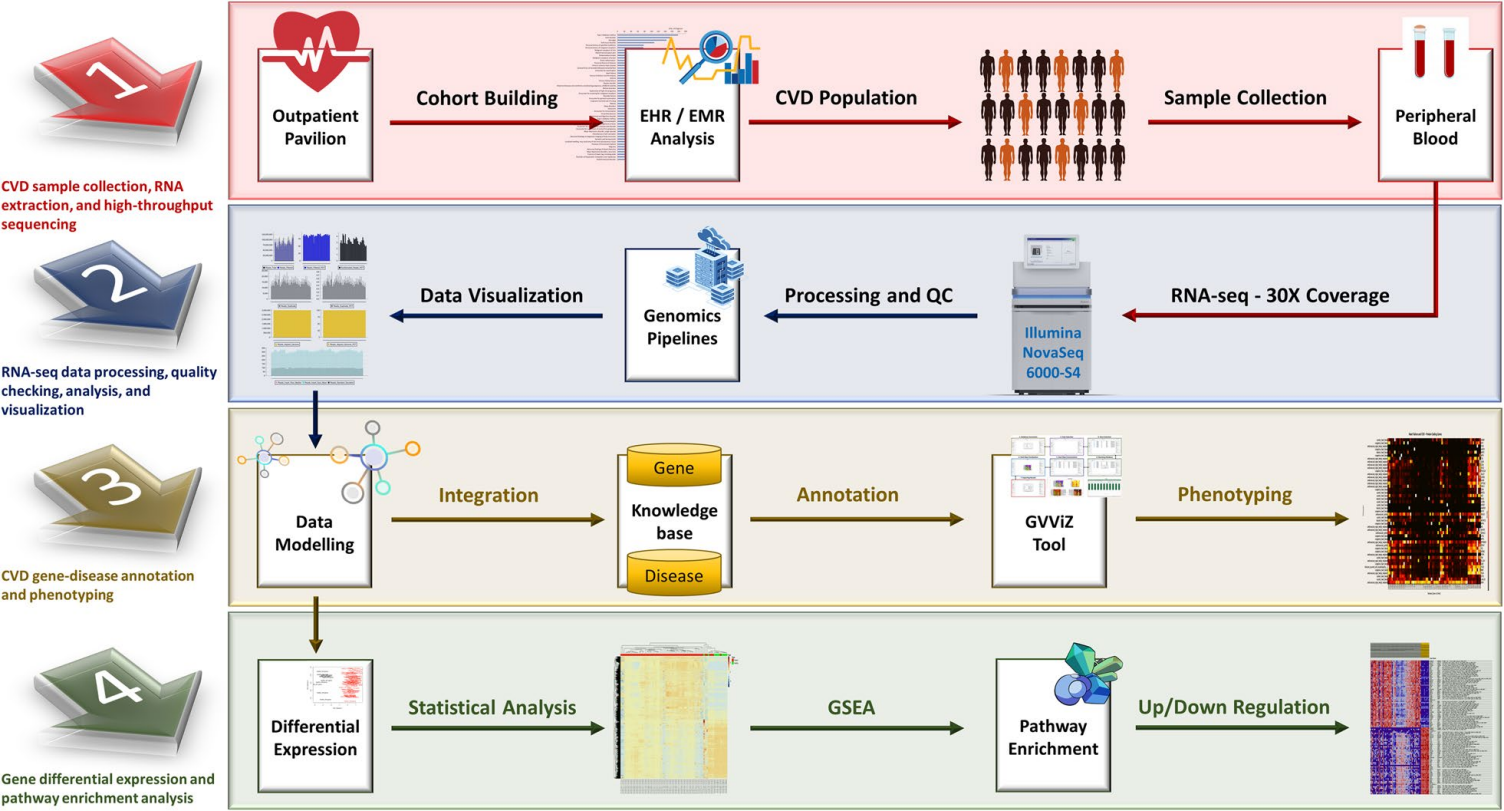
Research methodology divided among four major steps. Steps include, (1) CVD sample collection, RNA extraction, and high-throughput sequencing, (2) RNA-seq data processing, quality checking, analysis, and visualization, (3) CVD gene-disease annotation and phenotyping, and (4) Gene differential expression and pathway enrichment analysis

Overall study methodology is divided among four major steps, (1) CVD sample collection, RNA extraction, and high-throughput sequencing, (2) RNA-seq data processing, quality checking, analysis, and visualization, (3) CVD gene-disease annotation and phenotyping, and (4) gene differential expression and pathway enrichment analysis (Fig. 1).

### CVD sample collection, RNA extraction, and high-throughput sequencing

Supporting this study, we have developed an efficient data management system (PROMIS-LCR) for patient recruitment and consent, and for collecting, storing, and tracking of the original and current quantities of biospecimens under standardized conditions for preservation of critical metabolites. This system has been successfully deployed and is operational at the outpatient pavilion (OP) to support establishment of a biobank and a precision medicine initiative (PMI) at UConn Health. Highly heterogeneous and complex clinical terminologies have made electronic health records (EHRs) and diversified public content processing extremely arduous [28]. Addressing this challenge, we have developed an intelligent and dynamic data extract, transform, and loading (ETL) system for efficiently pulling clinical data from different health systems (EPIC and NextGen) and academic data models [29]. We implemented cutting-edge technologies utilizing artificial intelligence (AI) and machine learning (ML) approaches for multimodal data security, aggregation, classification, and examine granularities from population studies to subgroups stratification within the data continuum [28]. We investigated patient’s data centered on medical details, symptoms, age, race, gender, and demographics, and implemented healthcare data analytics process with features to build CVD cohort and from the population data [29]. This system, fully integrated with the PROMIS-LCR system, is tested and operational to efficiently extract and link de-identified medical details of the consented CVDs and even other patients participating in the PMI study with their collected biospecimens at UConn Health.

For high-throughput sequencing, peripheral blood was randomly extracted from 61 CVD patients. Table 1 presents details of all CVD patients (Sample IDs: 1059–1083) and that includes information about their gender (40 male and 21 female), ethnic groups (56 Not Hispanic, 4 Hispanic, and 1 Decline to Answer), and self-described race (42 Whites, 7 Blacks: Blacks or African Americans, 1 Asian, and 1 Decline to Answer, 2 other and 8 NA). These patients were clinically diagnosed with CVDs, and Systolic and Diastolic HF (CMS/HCC), including both heart failure with preserved ejection fraction (HFpEF) and heart failure with reduced ejection fraction (HFrEF). Additional reported diagnoses include cardiomyopathy, hypertension, obesity, type 2 diabetes mellitus, asthma, high cholesterol, hernia, chronic kidney, joint pain, myalgia, dizziness and giddiness, osteopenia of multiple sites, chest pain, and osteoarthritis. Built cohort is based on diverse individuals aged between 45 and 92. All ten healthy (control sample ids 648, 649, 650, 651, 652, 653, 655, 656, 657, 658) individuals (5 male and 5 female patients) had no clinical manifestation of any CVD and were aged between 28 and 78. Among control samples, three patients are self-described Hispanics (651, 656, 653), and the rest of the seven were categorized as non-Hispanic. Nine of them are from White race, and one was unknown (651). Further details are attached in the Additional file 1: Gender and age-based population data classification.

**Table 1:** Details of CVD sample details

CVD Sample IDs	Gender/Sex	Age	Ethnic groups	Race
1059	Male	79	Not_Hispanic	White
1068	Male	70	Not_Hispanic	NA
1073	Female	89	Not_Hispanic	White
1084	Female	69	Hispanic	Other
1085	Male	64	Hispanic	Other
1086	Male	65	Not_Hispanic	Black: Black or African American
1087	Female	69	Not_Hispanic	NA
1088	Female	65	Not_Hispanic	White
1089	Male	55	Not_Hispanic	White
1090	Male	70	Not_Hispanic	White
1091	Male	77	Not_Hispanic	White
1092	Male	62	Not_Hispanic	White
1093	Female	70	Not_Hispanic	White
1094	Male	64	Not_Hispanic	White
1095	Male	66	Not_Hispanic	White
1096	Male	59	Not_Hispanic	Black: Black or African American
1097	Female	57	Not_Hispanic	White
1098	Male	83	Not_Hispanic	NA
1099	Male	67	Not_Hispanic	White
1100	Male	81	Not_Hispanic	NA
1101	Male	64	Not_Hispanic	White
1102	Male	71	Not_Hispanic	Black: Black or African American
1103	Male	80	Not_Hispanic	White
1104	Male	73	Not_Hispanic	White
1105	Female	71	Not_Hispanic	White
1106	Male	79	Not_Hispanic	NA
1107	Male	84	Not_Hispanic	White
1108	Female	57	Not_Hispanic	Black: Black or African American
1109	Male	75	Not_Hispanic	White
1110	Male	80	Decline	Decline to Answer
1111	Female	86	Not_Hispanic	White
1112	Male	72	Hispanic	White
1113	Male	60	Hispanic	White
1114	Female	54	Not_Hispanic	Black: Black or African American
1115	Male	67	Not_Hispanic	White
1116	Female	63	Not_Hispanic	White
1117	Male	66	Not_Hispanic	White
1118	Male	88	Not_Hispanic	White
1058	Female	72	Not_Hispanic	White
1060	Male	58	Not_Hispanic	NA
1061	Male	70	Not_Hispanic	White
1062	Male	67	Not_Hispanic	White
1063	Male	66	Not_Hispanic	White
1064	Female	54	Not_Hispanic	NA
1065	Female	51	Not_Hispanic	White
1066	Male	82	Not_Hispanic	White
1067	Male	62	Not_Hispanic	White
1069	Female	65	Not_Hispanic	White
1070	Male	57	Not_Hispanic	White
1071	Female	52	Not_Hispanic	Asian
1072	Female	91	Not_Hispanic	White
1074	Female	81	Not_Hispanic	White
1075	Female	59	Not_Hispanic	White
1076	Male	45	Not_Hispanic	White
1077	Male	73	Not_Hispanic	White
1078	Female	72	Not_Hispanic	White
1079	Male	92	Not_Hispanic	NA
1080	Male	86	Not_Hispanic	White
1081	Male	57	Not_Hispanic	Black: Black or African American
1082	Female	59	Not_Hispanic	Black: Black or African American
1083	Male	85	Not_Hispanic	White

This table includes CVD Sample IDs (1059–1083), Gender/Sex (40 Male, and 21 Female), Age, Ethnic Groups (56 Not Hispanic, 4 Hispanic, and 1 Decline to Answer), and Race (42 White, 7 Black: Black or African American, 1 Asian, and 1 Decline to Answer, 2 other and 8 NA). NA = Not Available

Written informed consent was obtained from all subjects. All procedures performed in studies involving human participants were in accordance with the ethical standards of the institutional and with the 1964 Helsinki declaration and its later amendments or comparable ethical standards. All human samples were used in accordance with relevant guidelines and regulations, and all experimental protocols were approved by Institutional Review Board (IRB), UConn Health. Samples were curated, and all sequencing was done using the Illumina platform. Total RNA was extracted according to the manufacturer’s instructions. RNA quality was assessed for RNA integrity number. For all samples, RNA integrity number was > 7. An Illumina NovaSeq 6000-S4 was used for sequencing. An RNA Sample Preparation kit (Illumina, Inc.) was used for the preparation of cDNA libraries; cDNA libraries that passed size and purity check were retained for the following sequencing. Paired-end 150 bp short sequences (reads, pool across 2 lanes) with 30X coverage were generated for the blood samples, including the Illumina-compatible library (TruSeq Stranded mRNA).

### RNA-seq data processing, quality checking, analysis, and visualization

**Fig. 2:**
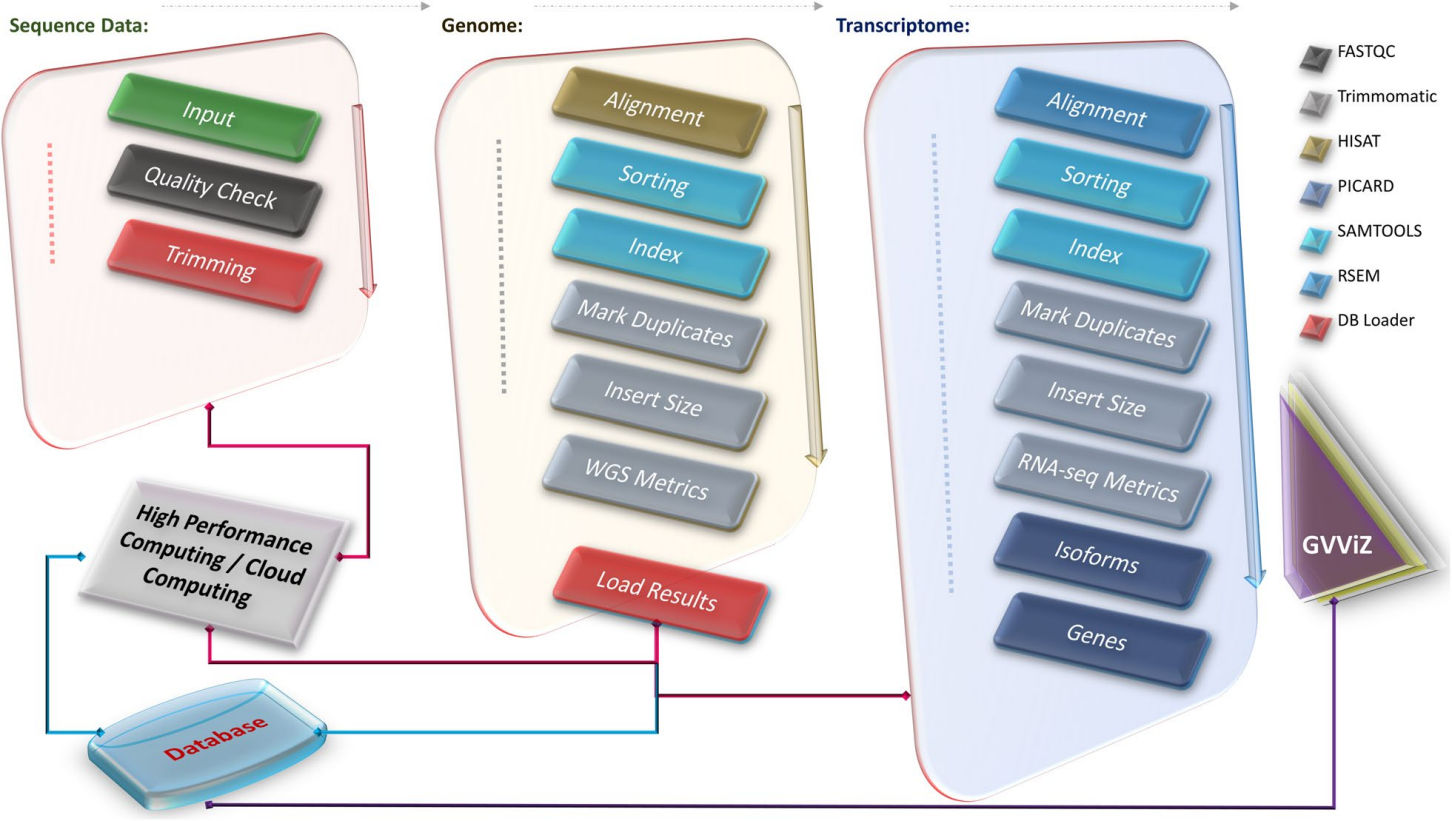
RNA-seq data processing pipeline. Used FastQC for quality checking; Trimmomatic to remove adapters and low-quality sequences; SAMtools to sort and index sequences; MarkDuplicates to remove duplicates; CollectInsertSizeMetrics to compute size distribution and read orientation of paired-end libraries; HISAT with Bowtie2 to align sequences to the human reference genome; and RSEM to quantify and identify differentially expressed genes by aligning reads to reference de novo transcriptome assemblies

To process and check the quality of RNA-seq data, we developed a pipeline with four operating modules: data pre-processing; data quality checking; data storage and management; and data visualization (Fig. 2). Quality control of raw reads was conducted using FastQC [30], which showed that all raw reads were qualified for downstream analysis. The reads were trimmed using Trimmomatic [31]. We used SAMtools for sorting sequences [32], MarkDuplicates for removing duplicates [33], and CollectInsertSizeMetrics by Picard to compute size distribution and read orientation of paired-end libraries. Afterward, the paired-end raw reads were aligned to the human reference genome (hg38) using HISAT [34] with Bowtie2 [35] software. RNA-seq by expectation maximization (RSEM) [26] was then applied for quantification and identification of identify differentially expressed genes (DEGs) by aligning reads to reference de novo transcriptome assemblies, based on transcript per million mapped reads (TPM). We used TPM as it is the best performing normalization method because it increases the proportion of variation attributable to biology compared to the raw data [36]. The decide-tests were performed to identify DEGs with Benjamini & Hochberg adjustment. Genes with *P* < 0.05 were selected as the criteria for significant differences (statistical values of all the DEGs are available in the Additional file 3: All DEGs Expression). Hierarchical clustering of DEGs was performed using the “pheatmap” function of the R/Bioconductor package. Expression analysis was also performed to see that the main source of variation is due to biological effects. This analysis was done on genes with an expression level higher than 50 TPM in at least one sample remained as high confidence genes (expression values of all the DEGs are available in the Additional file 5: All DEGs Stats 42 Genes). All computational results were stored in a designated database, using an in-house programmed command line data parser. The expression data were illustrated using the Gene Variant Visualization (GVViZ) environment, another bioinformatics application [37] developed in-house for efficient high-volume sequence data visualization.

### CVD gene-disease annotation and phenotyping

We have modelled and published a comprehensive knowledgebase of annotated disease-gene-variant data based on multiple clinical and genomics databases, including but not limited to ClinVar, GeneCards, MalaCard, DISEASES, HGMD, Disease Ontology, DiseaseEnhancer, DisGeNET, eDGAR, GTR, OMIM, miR2Disease, DNetDB, The Cancer Genome Atlas, International Cancer Genome Consortium, OMIM, GTR, CNVD, Ensembl, GenCode, Novoseek, Swiss-Prot, LncRNADisease, Orphanet, WHO, FDA, Catalogue Of Somatic Mutations In Cancer (COSMIC), and Genome-wide Association Studies (GWAS) [27, 38, 39]. We used this repository to perform gene-disease annotation for this study and found 43 genes associated with HF. They are *TNF, IL6, ACE, MMP2, NOS3, AGT, EDN1, REN, MYH7, AGTR1, AGTR1, NPPA, ADRB2, NR3C2, NR3C2, MME, CRP, MYH6, EPO, CST3, EDNRA, AQP2, MYBPC3, KNG1, VCL, HOTAIR, CDKN2B-AS1, ANKRD1, ADM, AMPD1, PLN, LGALS3, NPPB, ADRB1, UTS2, PIK3C2A, NPPC, CORIN, NPR1, LSINCT5, TUSC7, HSPB7,* and *RP11-451G4.2* (Table 2). Twenty-three genes associated with other CVDs phenotypes were: *SLC2A1, FGF2, FLNA, HBA1, GJB6, ATP2A2, CD40LG, FGF23, TEK, TAC1, DDX41, FADD, ENO2, LEMD3, CD34, TRPV1, GLMN, MB, SMUG1, PDPN, CALD1, KANTR, ZBTB8OS* (Table 3). Additional information about these genes is provided in Tables 1 and 2, including names, Ensembl ids, categories, diseases, and chromosomes.

**Table 2:** List of genes associated with the heart failure diseases

Gene names	Ensembl Ids	Categories	Diseases	Chromosomes	Regulation versus healthy controls
TNF	ENSG00000232810	Protein Coding	Systolic heart failure	chr6	Down
IL6	ENSG00000136244	Protein Coding	Systolic heart failure	chr7	Down
ACE	ENSG00000159640	Protein Coding	Congestive heart failure Diastolic heart failure Systolic heart failure	chr17	Down
MMP2	ENSG00000087245	Protein Coding	Diastolic heart failure	chr16	Down
NOS3	ENSG00000164867	Protein Coding	Diastolic heart failure	chr7	Down
AGT	ENSG00000135744	Protein Coding	Diastolic heart failure	chr1	Down
EDN1	ENSG00000078401	Protein Coding	Congestive heart failure	chr6	Down
REN	ENSG00000143839	Protein Coding	Congestive heart failure	chr1	Down
MYH7	ENSG00000092054	Protein Coding	Congestive heart failure	chr14	Up
AGTR1	ENSG00000144891	Protein Coding	Diastolic heart failure	chr3	Down
NPPA	ENSG00000175206	Protein Coding	Congestive heart failure Diastolic heart failure	chr1	Down
ADRB2	ENSG00000169252	Protein Coding	Congestive heart failure	chr5	Down
NR3C2	ENSG00000151623	Protein Coding	Congestive heart failure Systolic heart failure	chr4	Down
MME	ENSG00000196549	Protein Coding	Congestive heart failure	chr3	Down
CRP	ENSG00000132693	Protein Coding	systolic heart failure	chr1	Down
MYH6	ENSG00000197616	Protein Coding	Congestive heart failure	chr14	Down
EPO	ENSG00000130427	Protein Coding	Congestive heart failure	chr7	Down
CST3	ENSG00000101439	Protein Coding	Systolic heart failure	chr20	Down
EDNRA	ENSG00000151617	Protein Coding	Congestive heart failure	chr4	Down
AQP2	ENSG00000167580	Protein Coding	Congestive heart failure	chr12	Down
MYBPC3	ENSG00000134571	Protein Coding	Diastolic heart failure	chr11	Down
KNG1	ENSG00000113889	Protein Coding	Congestive heart failure	chr3	Down
VCL	ENSG00000035403	Protein Coding	Congestive heart failure	chr10	Down
HOTAIR	ENSG00000228630	antisense	Congestive heart failure	chr12	Down
CDKN2B-AS1	ENSG00000240498	antisense	Congestive heart failure	chr9	Down
ANKRD1	ENSG00000148677	Protein Coding	Diastolic heart failure	chr10	Up
ADM	ENSG00000148926	Protein Coding	Congestive heart failure	chr11	Down
AMPD1	ENSG00000116748	Protein Coding	Congestive heart failure	chr1	Up
PLN	ENSG00000198523	Protein Coding	Congestive heart failure	chr6	Down
LGALS3	ENSG00000131981	Protein Coding	Systolic heart failure	chr14	Down
NPPB	ENSG00000120937	Protein Coding	Congestive heart failure Diastolic heart failure Systolic heart failure	chr1	Down
ADRB1	ENSG00000043591	Protein Coding	Congestive heart failure Systolic heart failure	chr10	Down
UTS2	ENSG00000049247	Protein Coding	Congestive heart failure	chr1	Down
PIK3C2A	ENSG00000011405	Protein Coding	Congestive heart failure	chr11	Down
NPPC	ENSG00000163273	Protein Coding	Congestive heart failure	chr2	Up
CORIN	ENSG00000145244	Protein Coding	Systolic heart failure	chr4	Down
NPR1	ENSG00000169418	Protein Coding	Congestive heart failure	chr1	Up
LSINCT5	ENSG00000281560	lincRNA	Congestive heart failure	chr5	Down
TUSC7	ENSG00000243197	lincRNA	Congestive heart failure	chr3	Down
HSPB7	ENSG00000173641	Protein Coding	Systolic heart failure	chr1	Up
RP11-451G4.2	ENSG00000240045	Protein Coding	Heart failure	chr3	Down

**Table 3:** List of genes associated with the cardiovascular diseases

Gene names	Ensembl Ids	Categories	Diseases	Chromosomes	Regulation versus healthy controls
SLC2A1	ENSG00000117394	Protein Coding	Cardiovascular organ benign neoplasm	chr1	Down
FGF2	ENSG00000138685	Protein Coding	Cardiovascular organ benign neoplasm	chr4	Down
FLNA	ENSG00000196924	Protein Coding	Cardiovascular organ benign neoplasm	chrX	Down
HBA1	ENSG00000206172	Protein Coding	Cardiovascular organ benign neoplasm	chr16	Up
GJB6	ENSG00000121742	Protein Coding	Cardiovascular organ benign neoplasm	chr13	Down
ATP2A2	ENSG00000174437	Protein Coding	Cardiovascular organ benign neoplasm	chr12	Down
CD40LG	ENSG00000102245	Protein Coding	Cardiovascular syphilis	chrX	Down
FGF23	ENSG00000118972	Protein Coding	cardiovascular organ benign neoplasm	chr12	Down
TEK	ENSG00000120156	Protein Coding	Cardiovascular organ benign neoplasm	chr9	Down
TAC1	ENSG00000006128	Protein Coding	Cardiovascular organ benign neoplasm	chr7	Down
DDX41	ENSG00000183258	Protein Coding	Cardiovascular syphilis	chr5	Down
FADD	ENSG00000168040	Protein Coding	Infections recurrent with encephalopathy hepatic dysfunction and cardiovascular malformations	chr11	Down
ENO2	ENSG00000111674	Protein Coding	Cardiovascular organ benign neoplasm	chr12	Down
LEMD3	ENSG00000174106	Protein Coding	cardiovascular organ benign neoplasm	chr12	Down
CD34	ENSG00000174059	Protein Coding	cardiovascular organ benign neoplasm	chr1	Down
TRPV1	ENSG00000196689	Protein Coding	cardiovascular organ benign neoplasm	chr17	Down
GLMN	ENSG00000174842	Protein Coding	cardiovascular organ benign neoplasm	chr1	Down
MB	ENSG00000198125	Protein Coding	Cardiovascular organ benign neoplasm	chr22	Up
SMUG1	ENSG00000123415	Protein Coding	Cardiovascular syphilis	chr12	Up
PDPN	ENSG00000162493	Protein Coding	Cardiovascular organ benign neoplasm	chr1	Down
CALD1	ENSG00000122786	Protein Coding	Cardiovascular organ benign neoplasm	chr7	Down
KANTR	ENSG00000232593	Protein Coding	Cardiovascular organ benign neoplasm	chrX	Down
ZBTB8OS	ENSG00000176261	Protein Coding	Cardiovascular organ benign neoplasm	chr1	Down

### Gene differential expression and pathway enrichment analysis

To associate cellular functions with the DSGs, Gene Set Enrichment Analysis (GSEA) [40] was performed to verify the differences between comparisons. GSEA was carried out by using the curated gene sets of the Molecular Signature Database v7.0. The gene lists of hallmark gene sets (H), Kyoto Encyclopedia of Genes and Genomes (KEGG) pathway database (C2), and REACTOME pathway database (C2) were used to run GSEA, following the standard procedure described by GSEA user guide. Significantly enriched terms with similar descriptions and functions were further grouped into distinct biological categories (to better reflect the biology in context) and top categories were schematically projected on the network of enriched terms.

## Results

**Fig. 3:**
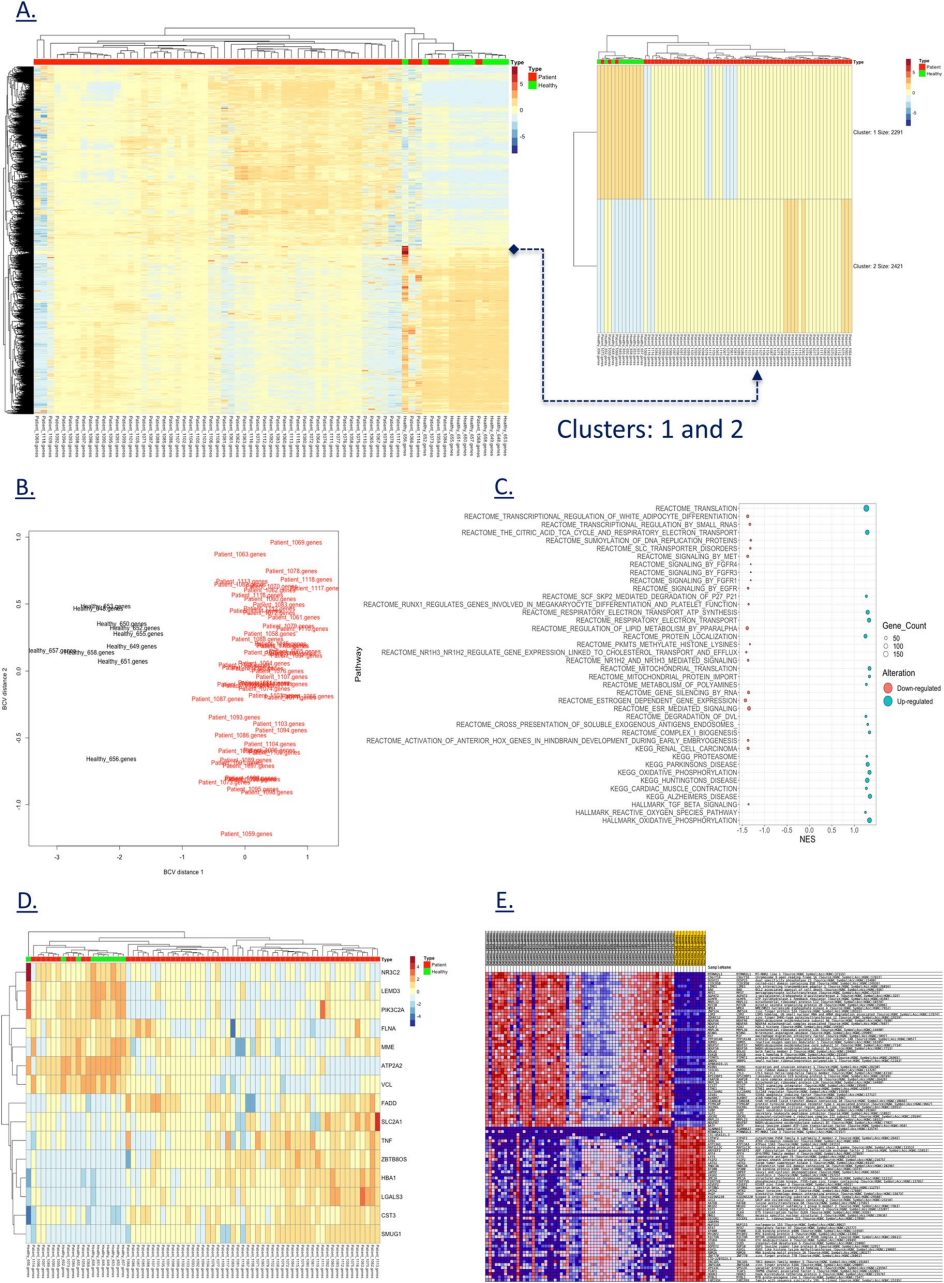
Differentially regulated gene expression and enrichment. **A** Differential gene expression of protein coding genes with two major clusters. **B** MDS plot showing biological distance between case–control samples based on BCV. **C** Top 20 enriched pathways showing up-regulation and down-regulation in CVD based on their normalized enrichment scores (NES). **D** Differential gene expression of annotated CVD genes. **E** Gene enrichment heatmap of differentially expressed genes

Cardiovascular disease is the most important cause of morbidity and mortality in developed countries, causing twice as many deaths as cancer in the USA. The underlying molecular pathogenic mechanisms for these disorders are still largely unknown, but gene expression may play a central role in the development and progression of cardiovascular disease. In this context, we have performed a comprehensive expression study comprising of two types of expression analysis between healthy controls and CVD patients diagnosed with HF and other cardiovascular phenotypes. We started with a global differential gene expression analysis based on TPM count for protein genes to identify significantly differentiated genes (Fig. 3A). We generated a multidimensional scaling (MDS) [41] plot of biological coefficient of variation (BCV) [42] to identify biological variation between case and control groups (Fig. 3B). There were no outliers seen in the MDS plot. We identified 4,712 DEGs between the controls and the CVD group (Fig. 3A) which can be grouped into two clusters (kmeans row clustering) (Fig. 3A). Statistical significance of P value < 0.05 and |log2FC| ≥ 2 showed 42 genes with greater than twofold change. Some of these highly significant genes have already been reported in multiple CVDs (APOD, PIGR, CELSR1, COBLL1, FCRL5, TEAD2, ABCA6, COL4A3, CYP4F2, FMOD, GNG8, IGF2R, PEG10, RAPGEF3, RASGRF1, SCARNA17, TCF4), while some genes (ADAM29, ARHGAP44, CD200, CLEC17A, CLNK, CNTNAP1, CNTNAP2, CTC-454I21.3, DMD, FAM129C, FAM3C, FCRL1, FCRL2, FCRLA, GPM6A, KLHL14, MTRNR2L3, NPIPB5, OSBPL10, PAX5, PCDH9, PHYHD1, POU2AF1, RALGPS2, ZNF888) have shown a novel expression in CVD. Statistical difference in expression for these genes can be seen in the Additional file 4: All DEGs Stats. Gene enrichment of all the DEGs revealed 190 pathways upregulated in the CVD patients and 408 pathways were found to be down-regulated (Fig. 3E). Figure 3C shows top 20 up-regulated and down-regulated pathways in CVD patients. Major up-regulated pathways were protein translation and localization, cardiac muscle contraction, oxidative phosphorylation, mitochondrial translation and protein import, electron transport and citric acid cycle. The pathways involved in down-regulation included FGFR1, FGFR2, FGFR3, EGFR, TGF beta, MET mediated signaling, estrogen-dependent gene expression, NR1H2, NR1H3 mediated cholesterol transport and efflux, and regulation of white adipocytes differentiation. By default, gene sets are ordered by normalized enrichment score (NES). More details on all the enriched pathways are available in the Additional file 6: CVD Enrichments. From the list of annotated CVD genes, 15 genes showed a differentiated expression (Fig. 3D). Among them, 7 are HF genes (CST3, LGALS3, MME, NR3C2, PIK3C2A, TNF, VCL), and 8 are other CVD genes (ATP2A2, FADD, FLNA, HBA1, LEMD3, SLC2A1, SMUG1, ZBTB8OS). Enrichment of these genes showed down-regulation was seen in NR3C2, LEMD3, PIK3C2A, FLNA, MME, ATP2A2, and VCL, while a pattern of upregulation was observed in FADD, SLC2A1, TNF, ZBTB8OS, HBA1, LGALS3, CST3, and SMUG1, suggesting that intrinsic biological differences account for, at least, part of CVD.

**Fig. 4:**
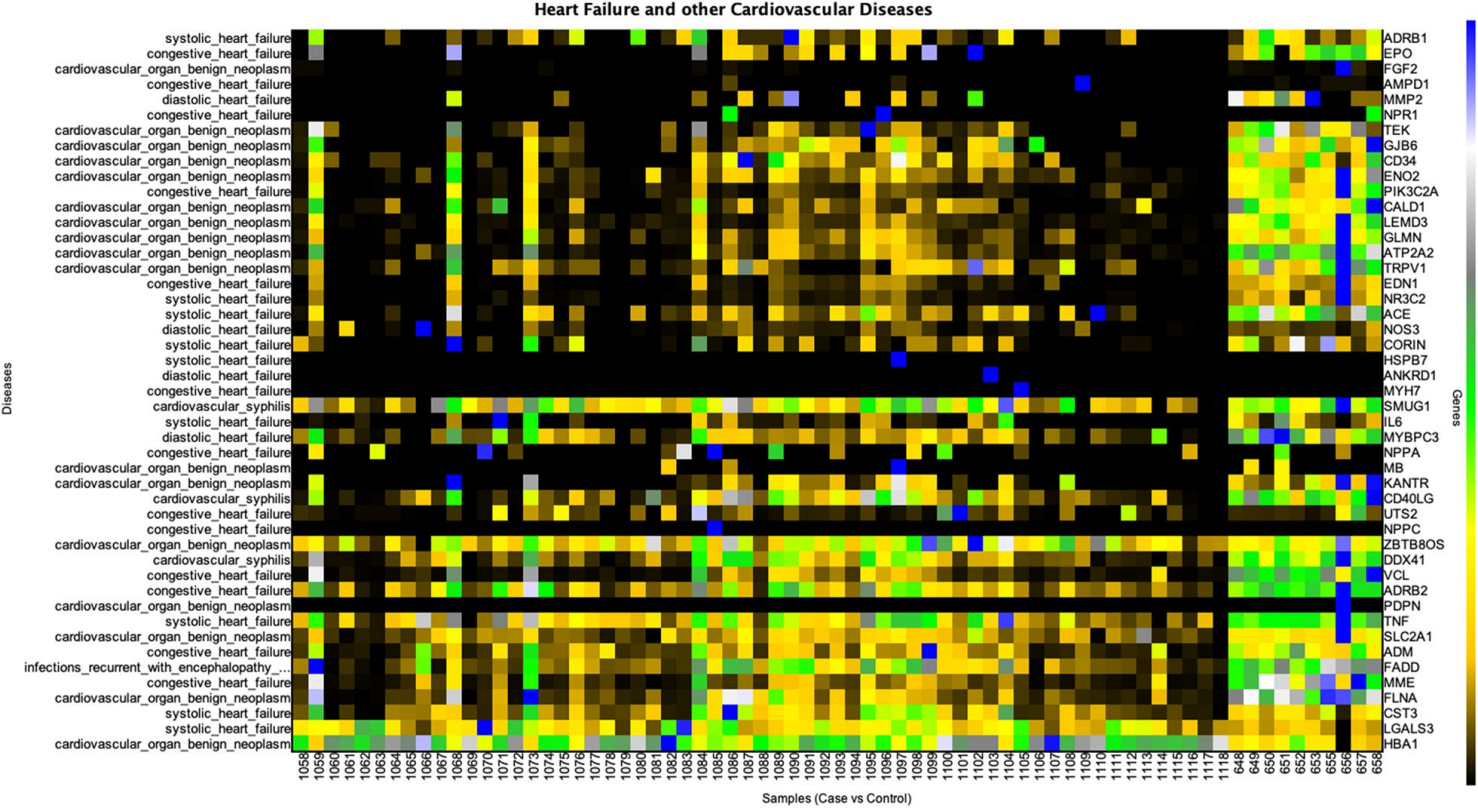
Gene expression analysis of all CVD genes. Genes-disease heatmap for the expression analysis of CVDs among all diseased and healthy control patients. The X-axis signifies samples (healthy ids: 648, 650, 651, 652, 653, 655, 656, and CVD ids: 1058–1118), the right Y-axis shows genes, and the left Y-axis presents genes associated with the CVDs

The second type of analysis was based on expression analysis to compare expression of all 48 CVD genes between CVD patients and healthy controls. We used our in-house developed GVViZ platform to perform expression analysis using TPM counts of the protein coding genes computed from RNA-seq data. Furthermore, the expression data were linked to gene-disease annotation databases [27, 38, 39] to classify and differentiate between CVD and other disease-based functional and non-functional genes. A heatmap of all the CVD genes was constructed (Fig. 4) and annotated with their associated clinical CVD phenotype. In GVViZ-generated Fig. 4, the X-axis signifies samples (healthy ids: 648, 649, 650, 651, 652, 653, 655, 656, 657, 658, and CVD ids: 1058–1118), the right Y-axis shows genes, and the left Y-axis presents genes associated with the CVDs. There were apparent differences in the filtered expression counts for healthy controls and CVD patients mapped to visualize the variations across the cohort. The analysis showed clear separation of a subset of CVD patients with significantly variable expression for a cluster of genes (details attached in the Additional file 7: Original Raw Data).

**Fig. 5:**
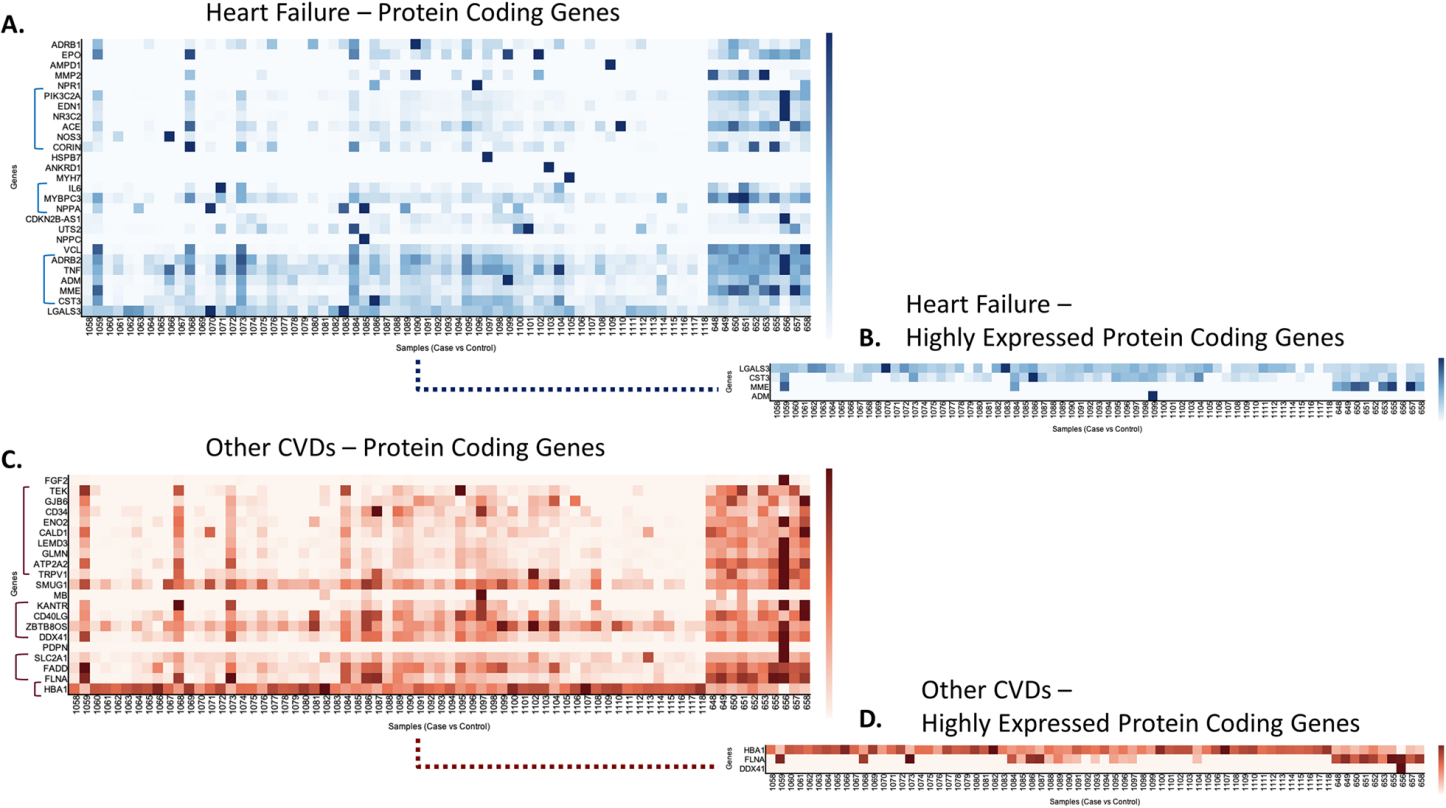
Gene expression analysis of HF and other CVD genes. **A** Expression analysis of protein-coding genes in HF. **B** Highly expressed protein-coding genes related to HF disease. **C** Expression analysis of protein-coding genes in other CVD genes. **D** Highly expressed protein-coding genes related to other CVDs

**Fig. 6:**
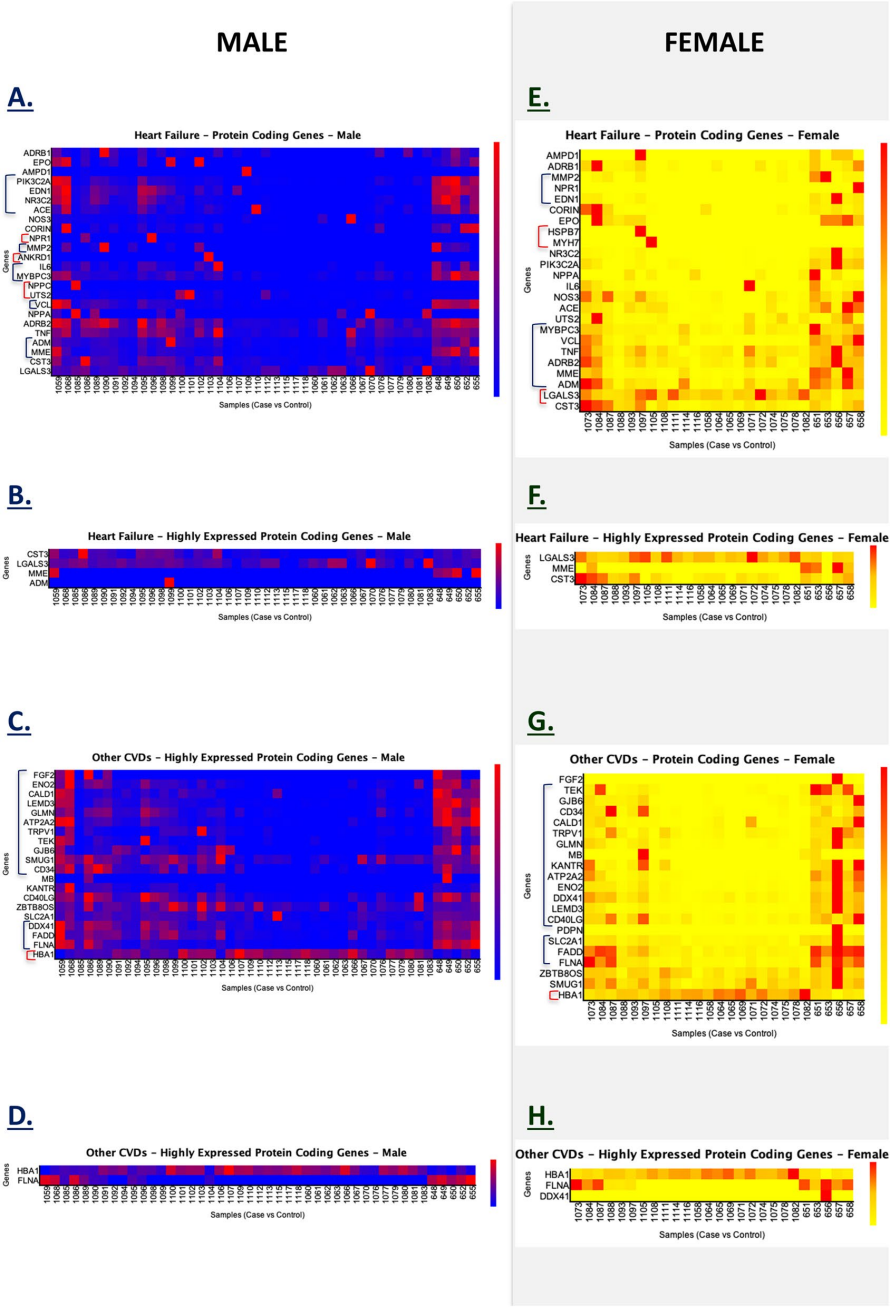
Gender-based gene expression analysis of HF and other CVD genes. **A** Protein-coding genes related to HF in males, **B** Highly expressed protein-coding genes related to HF in males, **C** Protein-coding genes related to CVD in males, **D** Highly expressed protein-coding genes related to CVD in males. **E** Protein-coding genes related to HF in females, **F** Highly expressed protein-coding genes related to HF in females, **G** Protein-coding genes related to other CVD sin females, and **H** highly expressed protein-coding genes related to other CVDs in females

To systematically inspect gene expression in this dataset, CVD patients were mainly stratified into condition, control, and gender for further analysis (Figs. 5 and 6). With a focus on HF and all other CVDs grouped together, we analyzed the expression of all protein coding genes (Fig. 5A), and only highly expressed protein-coding genes (Fig. 5B) related to HF disease, as well as expression analysis of protein coding genes (Fig. 5C), and only highly expressed protein coding genes (Fig. 5D) related to other CVDs. In GVViZ-generated Fig. 5, the X-axis signifies samples (healthy patient ids 648, 649, 650, 651, 652, 653, 655, 656, 657, 658; CVD patient ids 1058–1118), and the Y-axis shows genes associated with HF (Fig. 5A, B) and CVDs (Fig. 5C, D).

During this disease stratification (Fig. 5), we found patterns that significantly differentiate the HF and CVD groups from the healthy control group. Three clusters were identified in the HF expression analysis, which showed altered expression between the condition and the control groups (Fig. 5A). The first cluster consisted of five genes (*ADRB2*, *TNF*, *ADM*, *MME*, and *CST3*), the second cluster included three genes (*IL6*, *MYBPC3, NPPA*), and the third cluster contained seven genes (*PIK3C2A*, *EDN1*, *NR3C2*, *NMP2*, *ACE*, *NOS3*, and *CORIN*). Among these three clusters, all HF genes showed low expression compared to the healthy control group, indicating their down regulation. However, four HF protein-coding genes (*LGALS3*, *CST3*, *MME*, and *ADM*) showed high expression in one or more patients (Fig. 5B).

Expression analysis of genes accounting for other CVDs showed four clusters between healthy and disease groups (Fig. 5C). The first cluster included nine genes (*TEK*, *GJB6*, *CD34*, *ENO2*, *CALD1*, *LEMD3*, *GLMN*, *ATP2A2*, and *TRPV1*), the second cluster showed four genes (*KANTR*, *CD40LG*, *ZBTB8OS*, and *DDX41*), the third cluster consisted of three genes (*SLC2A1*, *FADD*, and *FLNA*), and the fourth cluster had only one gene (*HBA1*). Genes in the first cluster had over 80% of patients showing low expression in comparison with the healthy control group, indicating their down regulation. However, genes in the second and third clusters had over 50% patients with low expression compared to the control group. On the contrary, *HBA1* showed high expression during analysis. Other CVD protein-coding genes that had the highest expressed were *HBA1*, *FLNA*, and *DDX41* (Fig. 5D).

To further classify the groups, we performed gender-based gene expression analysis of HF and other CVD genes (Fig. 6). We compared gender-matched case and control groups (male CVD vs male controls, and female CVD vs female controls). The results illustrated for HF protein-coding genes in the male group (Fig. 6A, B) with genes showing a relatively low expression in comparison with the control group (*ADM*, *MME*, *VCL*, *MYBPC3*, *IL6*, *MMP2*, *ACE*, *NR3C2*, *EDN1*, and *PIK3C2A*). Some genes showed a rise in expression in comparison with the control group (*NPR1*, *ANKRD1*, *NPPC*, and *UTS2*). Looking at the HF protein-coding genes in the female group (Fig. 6E, F), gene *LGALS3* was found to be highly regulated among diseased samples in comparison with healthy controls, whereas some genes showed a down regulated expression (*ADM*, *MME*, *ADRB2*, *TNF*, *VCL*, *MYBPC3*, *MYH7*, *HDPB7*, *MMP2*, *NPR1,* and *EDN1*). Interestingly similar protein-coding genes related to HF were found to be highly expressed in both males and females (*CST3, LGALS3*, *MME)*. However, *ADM* was only found in males.

Likewise, gender-based gene expression analysis of other CVD genes revealed altered expression in the male group (Fig. 6C, D). We identified several CVD genes with low expression in the male cohort (*ELNA*, *FADD*, *DDX41*, *CD34*, *SMUG1*, *GJB6*, *TEK*, *TRPV1*, *ATP2A2*, *GLMN*, *LEMD3*, *CALD1*, *ENO2*, and *FGF2*). In the female group, we also observed low expression in CVD genes (*FLNA*, *FADD*, *SLC2A1*, *CD40LG*, *LEMD3*, *DDX41*, *ENO2*, *ATP2A2*, *KANTR*, *MB*, *GLMN*, *TRPV1*, *CALD1*, *CD34*, *GJB6*, *TEK*, and *FGF2*) (Fig. 6G, H). *HBA1*, *FLNA*, and *DDX41* were found as the highly expressed protein-coding CVD genes in both gender groups, and *ENO2* was the only highly expressed gene in the female group.

**Fig. 7:**
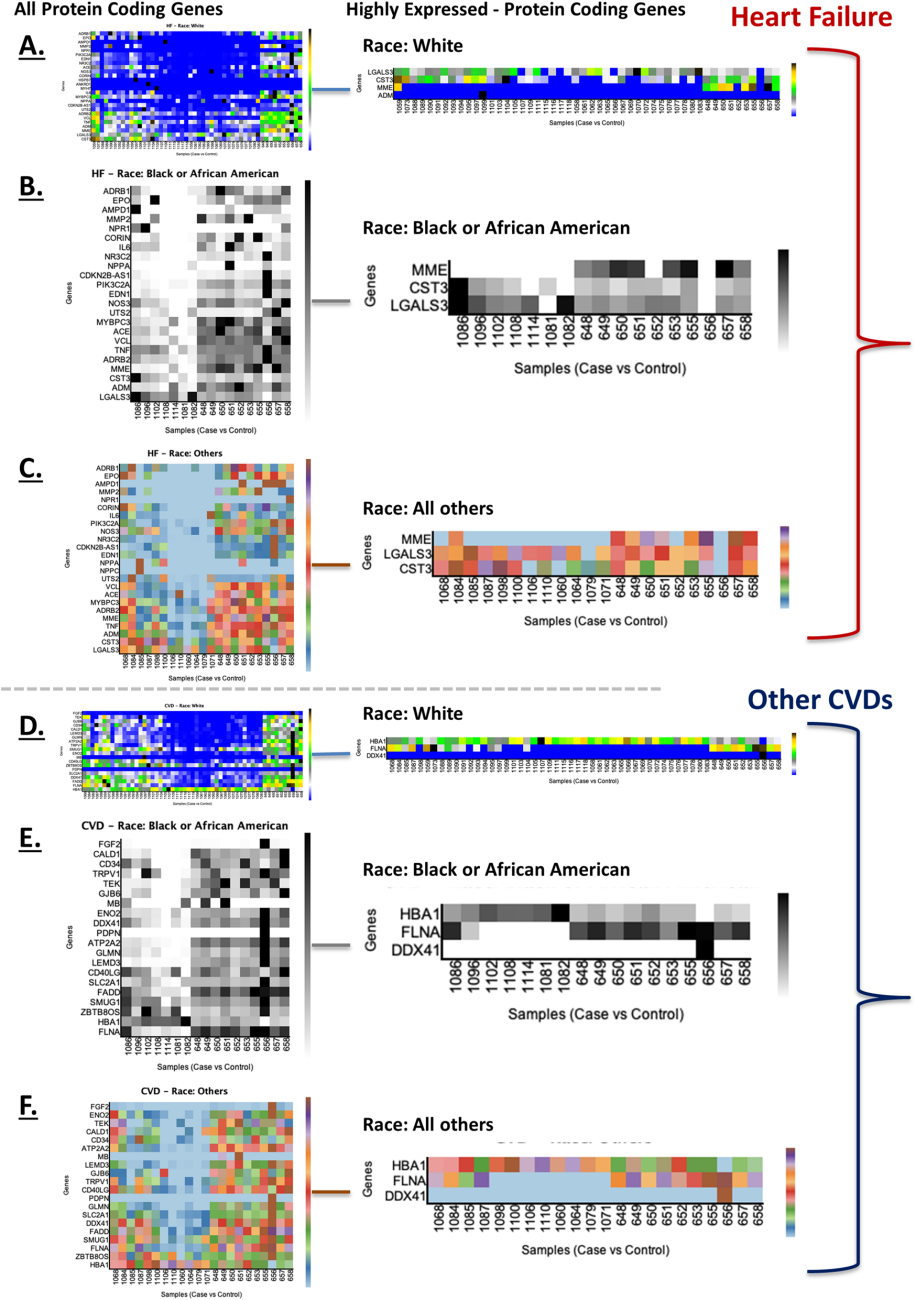
Race-based gene expression analysis of HF and other CVD genes. All and highly expressed protein-coding genes related to HF in self-described Whites (**A**), Blacks/African Americans (**B**), and all other races (**C**). All and highly expressed protein-coding genes related to other CVDs in Whites (**D**), Blacks/African Americans (**E**), and all other races (**F**)

We investigated HF and other CVD associated protein coding genes and their expression levels among difference races (Fig. 7). We observed MME, CST3 and LGALS3 HF genes with high expression among White Americans (Fig. 7A), Blacks/African Blacks (Fig. 7B), and all other races (Fig. 7C). When ADM was only located within White Americans. We commonly found DDX41, FLNA and HB1 CVD genes with high expression among white Americans (Fig. 7D), Blacks/African Blacks (Fig. 7E), and all other races (Fig. 7F). However, we have also presented all differentially expressed HF and other CVD genes among these all races in Fig. 7. High resolution figures are attached in Additional file 2. To incorporate produced results in clinical settings, and to get given recommendations back into EHRs, we have linked HF and other CVD genes (Ensembl) with the International Classification of Disease (ICD) codes (Table 4).

**Table 4:** List of heart failure (HF) and other CVD genes linked to ICD codes

Genes	Diseases	Ensembl Ids	ICD 10 codes
SLC2A1	CVD	ENSG00000117394	D15.1
FGF2	CVD	ENSG00000138685	D15.1
FLNA	CVD	ENSG00000196924	D15.1
HBA1	CVD	ENSG00000206172	D15.1
GJB6	CVD	ENSG00000121742	D15.1
ATP2A2	CVD	ENSG00000174437	D15.1
CD40LG	CVD	ENSG00000102245	A52.00
FGF23	CVD	ENSG00000118972	D15.1
TEK	CVD	ENSG00000120156	D15.1
TAC1	CVD	ENSG00000006128	D15.1
DDX41	CVD	ENSG00000183258	A52.00
FADD	CVD	ENSG00000168040	D53.0
ENO2	CVD	ENSG00000111674	D15.1
LEMD3	CVD	ENSG00000174106	D15.1
CD34	CVD	ENSG00000174059	D15.1
TRPV1	CVD	ENSG00000196689	D15.1
GLMN	CVD	ENSG00000174842	D15.1
MB	CVD	ENSG00000198125	D15.1
SMUG1	CVD	ENSG00000123415	A52.00
PDPN	CVD	ENSG00000162493	D15.1
CALD1	CVD	ENSG00000122786	D15.1
KANTR	CVD	ENSG00000232593	D15.1
ZBTB8OS	CVD	ENSG00000176261	D15.1
TNF	HF	ENSG00000232810	I50.20
IL6	HF	ENSG00000136244	I50.20
ACE	HF	ENSG00000159640	I50.9
ACE	HF	ENSG00000159640	I50.3
ACE	HF	ENSG00000159640	I50.20
MMP2	HF	ENSG00000087245	I50.3
NOS3	HF	ENSG00000164867	I50.3
AGT	HF	ENSG00000135744	I50.3
EDN1	HF	ENSG00000078401	I50.9
REN	HF	ENSG00000143839	I50.9
MYH7	HF	ENSG00000092054	I50.9
AGTR1	HF	ENSG00000144891	I50.3
AGTR1	HF	ENSG00000144891	I50.9
NPPA	HF	ENSG00000175206	I50.9
ADRB2	HF	ENSG00000169252	I50.9
NR3C2	HF	ENSG00000151623	I50.9
NR3C2	HF	ENSG00000151623	I50.20
MME	HF	ENSG00000196549	I50.9
CRP	HF	ENSG00000132693	I50.20
MYH6	HF	ENSG00000197616	I50.9
EPO	HF	ENSG00000130427	I50.9
CST3	HF	ENSG00000101439	I50.20
EDNRA	HF	ENSG00000151617	I50.9
AQP2	HF	ENSG00000167580	I50.9
MYBPC3	HF	ENSG00000134571	I50.3
KNG1	HF	ENSG00000113889	I50.9
VCL	HF	ENSG00000035403	I50.9
HOTAIR	HF	ENSG00000228630	I50.9
CDKN2B-AS1	HF	ENSG00000240498	I50.9
ANKRD1	HF	ENSG00000148677	I50.3
ADM	HF	ENSG00000148926	I50.9
AMPD1	HF	ENSG00000116748	I50.9
PLN	HF	ENSG00000198523	I50.9
LGALS3	HF	ENSG00000131981	I50.20
NPPB	HF	ENSG00000120937	I50.9
NPPB	HF	ENSG00000120937	I50.3
NPPB	HF	ENSG00000120937	I50.20
ADRB1	HF	ENSG00000043591	I50.9
ADRB1	HF	ENSG00000043591	I50.20
UTS2	HF	ENSG00000049247	I50.9
PIK3C2A	HF	ENSG00000011405	I50.9
NPPC	HF	ENSG00000163273	I50.9
CORIN	HF	ENSG00000145244	I50.20
NPR1	HF	ENSG00000169418	I50.9
LSINCT5	HF	ENSG00000281560	I50.9
TUSC7	HF	ENSG00000243197	I50.9
HSPB7	HF	ENSG00000173641	I50.20
RP11-451G4.2	HF	ENSG00000240045	I50.9

## Discussion

Over the past few years, genomic-sequencing technologies have emerged to improve the clinical diagnosis of genetic disorders and continuing to expand the potential of basic sciences in developing biological insights of human genetic variations and their biologic consequences [43]. Several clinically established cardiovascular circulating biomarkers are measured to help diagnose, stratify risk, and monitor people with suspected CVDs. Use of one or more of these biomarkers can help physicians identify a heart condition and initiate appropriate therapy, as well as follow the course of disease. CVD presents differently in women and men both symptomatically and biochemically [44]. However, some studies have failed to detect a heart condition in women with elevated death rates [45]. Lack of gender-specific cardiac biomarker thresholds in men and women may be the reason for CVD underdiagnosis in women, and potentially increased morbidity and mortality as a result, or conversely, an overdiagnosis in men.

Here, we report a peripheral blood gene expression analysis focused on HF- and CVD genes to identify gender-specific differences in patients aged between 45 and 95 years old. Our major findings include disease specific up- and down-regulated differentially expressed protein-coding genes in HF and CVDs and categorized their major signaling pathways involved in disease physiology. This analysis also revealed 25 novel gene expression in CVD patients. Our results on gender-specific differences in expression of protein-coding genes related to HF and other CVDs show that it is important to systematically investigate gender-differences in high-impact genes in HF and CVDs [46, 47]. We found differentially altered expression of *FLNA*, *CST3*, *LGALS3*, and *HBA1*, potentially responsible for HF and other CVDs in both male and female populations. *FLNA* is a gene known for CVDs, as mutations in *FLNA* can lead to cardiological phenotypes with aortic or mitral regurgitation [48]. High expression and mutations in the *CST3* (Cystatin C) gene have been reported in systolic HF, ischemic stroke, and CAD [49, 50]. The *LGALS3* gene encodes the galectin-3 (35-kDa) protein, and single nucleotide polymorphisms (SNPs) and promoter-regulated expression of *LGALS3* are considered potential candidates that cause CVDs, especially CAD, dilated cardiomyopathy, and HF [51–54]. The *HBA1* (glycated hemoglobin A1c) gene (chromosome 16) is considered a prognostic marker responsible for the increased cardiovascular mortality risk in age- and gender-classified populations [55, 56]. Mutations in *HBA1* can cause myocardial infarction, stroke, coronary heart disease, and HF [56]. The differential expression of *ENO2* (Enolase 2) gene in CVDs also highlighted gender-specific (female) alterations, which has been reported in other conditions [57].

RNA-seq driven gene expression analysis is an advancement in the field clinical genomics to analyze chromatin and patterns of expression in genes and differentiating the pathways, which differ between healthy and diseased people [43]. Our study aimed to investigate the clinical significance of gene expression in HF and CVDs using RNA-seq data. We analyzed the differences between healthy and diseased states to understand the pathology of disease [58]. The risk for and the course of heart failure also depends on genomic variants and mutations underlying the so‐called genetic predisposition. Several studies have demonstrated that only about half of all DNA genetic variants are detectable by RNA sequencing of human tissue and cell lines [59–61]. However, this approach has some potential limitations. Accurate capture of DNA variants using the RNA-seq data requires high coverage and sufficient samples per population as it has already been tested in cancer [62, 63], which we expect will be mitigated by generating whole genome sequencing (WGS) data to perform variant analysis of the genes responsible for HF (Table 2) and CVDs (Table 3). Nonetheless, with a need to expand the cohort of healthy controls to investigate DEGs with significantly regulated expression and increase the power to substantiate association with related variables in the CVD populations will help to scale down to clinically important genetic variations. Also, PCR validation of the differentially regulated genes will add prognostic value to the study and consolidate the role of specific genes as important biomarkers in HF. Our future plans involve application of AI and ML techniques [28] to advance investigating correlation and overlapping of reported diagnoses of HF and CVD patients in clinical data. Finally, assessment of genotype and phenotype associations to find potentially high-risk indistinct results for patient care from highly regulated genes and disease-causing variants [11].

## Conclusion

Our analysis identified four altered expression of HF- and other CVD genes (FLNA, CST3, LGALS3, and HBA1) with gender differences in middle-aged to frail patients and revealed differential regulation of 41 genes related to HF and 23 genes related to other CVDs. Furthermore, many pathways were found to be enriched, and gender-specific analysis showed shared and unique genes between the genders. Additional testing of these genes may lead to the development of new clinical tools to improve diagnosis and prognosis of CVD patients.

## Supplementary Information

**Additional file 1.** Gender and age-based population data classification.

**Additional file 2.** High resolution figures.

**Additional file 3.** All DEGs Expression

**Additional file 4.** All DEGs Stats

**Additional file 5.** All DEGs Stats 42 Genes

**Additional file 6.** CVD Enrichments

**Additional file 7.** Original Raw Data

## Data Availability

The data analyzed in the current study are available from the corresponding author on reasonable request.

## Abbreviations

AMI: Acute myocardial infarction
AI: Artificial intelligence
BCV: Biological coefficient of variation
CVDs: Cardiovascular diseases
COSMIC: Catalogue of somatic mutations in cancer
CDC: Center for disease control and prevention
DEGs: Differentially expressed genes
HER: Electronic health record
EMR: Electronic medical record
EM: Expectation–maximization
ETL: Extract, transform, and loading
GWAS: Genome-wide Association Studies
GVViZ: Gene variant visualization
GSEA: Gene set enrichment analysis
HF: Heart failure
HFpEF: Heart failure with preserved ejection fraction
HFrEF: Heart failure with reduced ejection fraction
hg38: Human reference genome
ICD: International Classification of Disease codes
KEGG: Kyoto Encyclopedia of Genes and Genomes
ML: Machine learning
mRNAs: Messenger ribonucleic acids
MDS: Multidimensional scaling
OP: Outpatient pavilion
RNA-seq: RNA sequencing
RSEM: RNA-seq by expectation maximization
CMS/HCC: Systolic and diastolic HF
TPM: Transcript per million mapped reads
WGS: Whole genome sequencing
