# Supplementary material for: RNA-seq driven expression and enrichment analysis to investigate CVD genes with associated phenotypes among high-risk heart failure patients
Source: Hum Genomics. 2021 Nov 13;15:67. doi: 10.1186/s40246-021-00367-8 (PMC8590246; doi:10.1186/s40246-021-00367-8)
Supplement: Supplementary file 1 — Additional file 1. Gender and age-based population data classification. [file 40246_2021_367_MOESM1_ESM.pdf]

Supplementary material 1:

Gender and age-based population data classification.

Title

RNA-seq driven expression and enrichment analysis to investigate CVD genes with associated phenotypes among high-risk Heart Failure patients.

Authors

Zeeshan Ahmed[1,2,4,5*], Saman Zeeshan[3], and Bruce T. Liang[5]

Affiliations

1. Rutgers Institute for Health, Health Care Policy and Aging Research, Rutgers University, 112 Paterson St, New Brunswick, NJ, USA.

2. Department of Medicine, Robert Wood Johnson Medical School, Rutgers Biomedical and Health Sciences, 125 Paterson St, New Brunswick, NJ, USA.

3. Rutgers Cancer Institute of New Jersey, Rutgers University, 195 Little Albany St, New Brunswick, NJ, USA.

4. Department of Genetics and Genome Sciences, UConn Health, 400 Farmington Ave, Farmington, CT, USA.

5. Pat and Jim Calhoun Cardiology Center, UConn School of Medicine, University of Connecticut Health Center, 263 Farmington Ave, Farmington, CT, USA.

[*]Corresponding author: Zeeshan Ahmed, Rutgers Institute for Health, Health Care Policy and Aging Research, Rutgers University, 112 Paterson Street, New Brunswick, 08901, NJ, USA. (zahmed@ifh.rutgers.edu and zahmed@uchc.edu).

Supplementary material 1:

Gender and age-based population data classification.

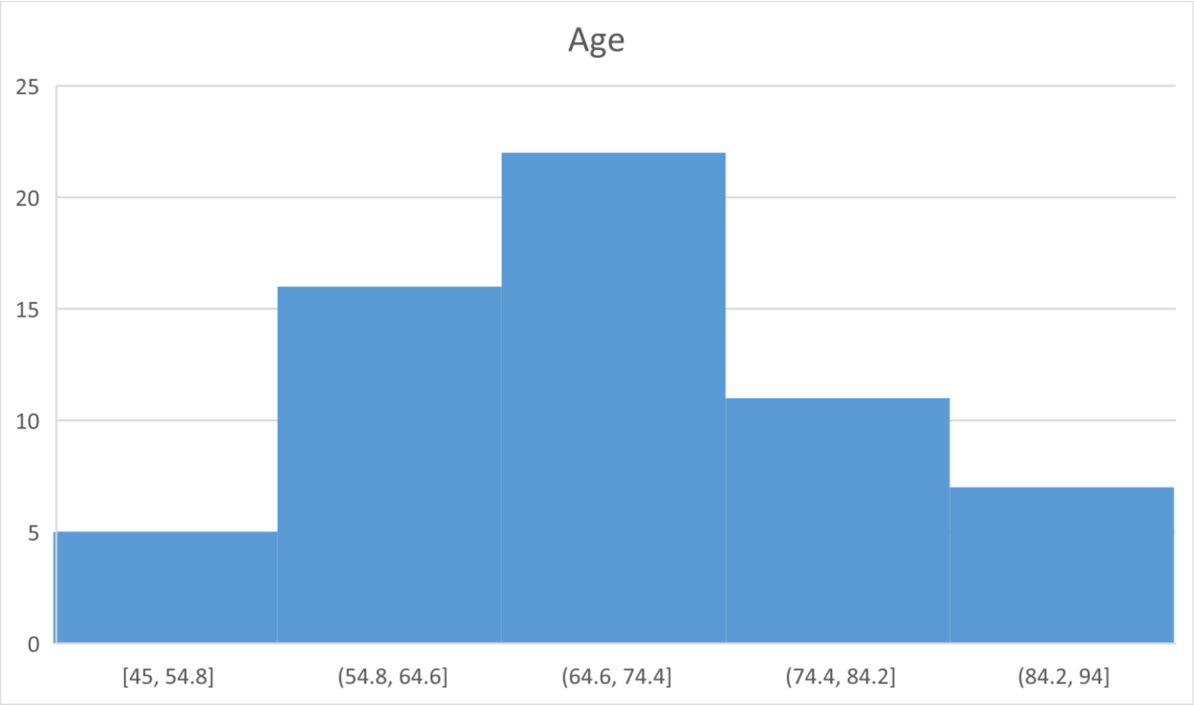

S1. Figure 1: Gender and age-based population data classification – Age Female.

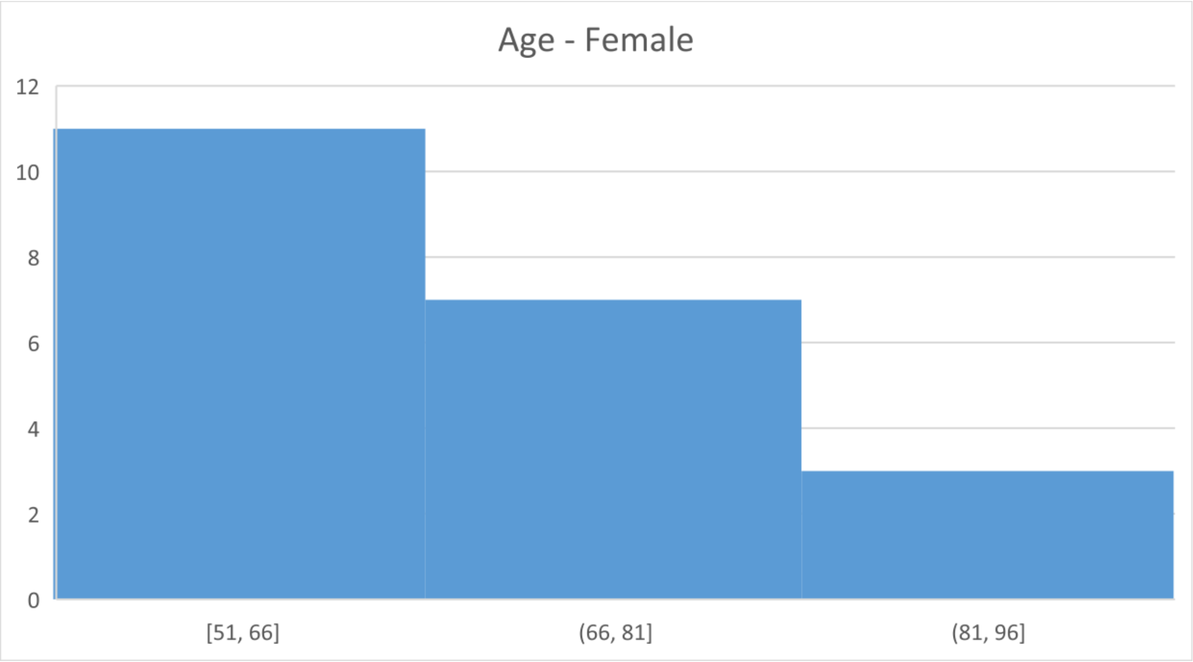

S1. Figure 2: Gender and age-based population data classification – Age Female.

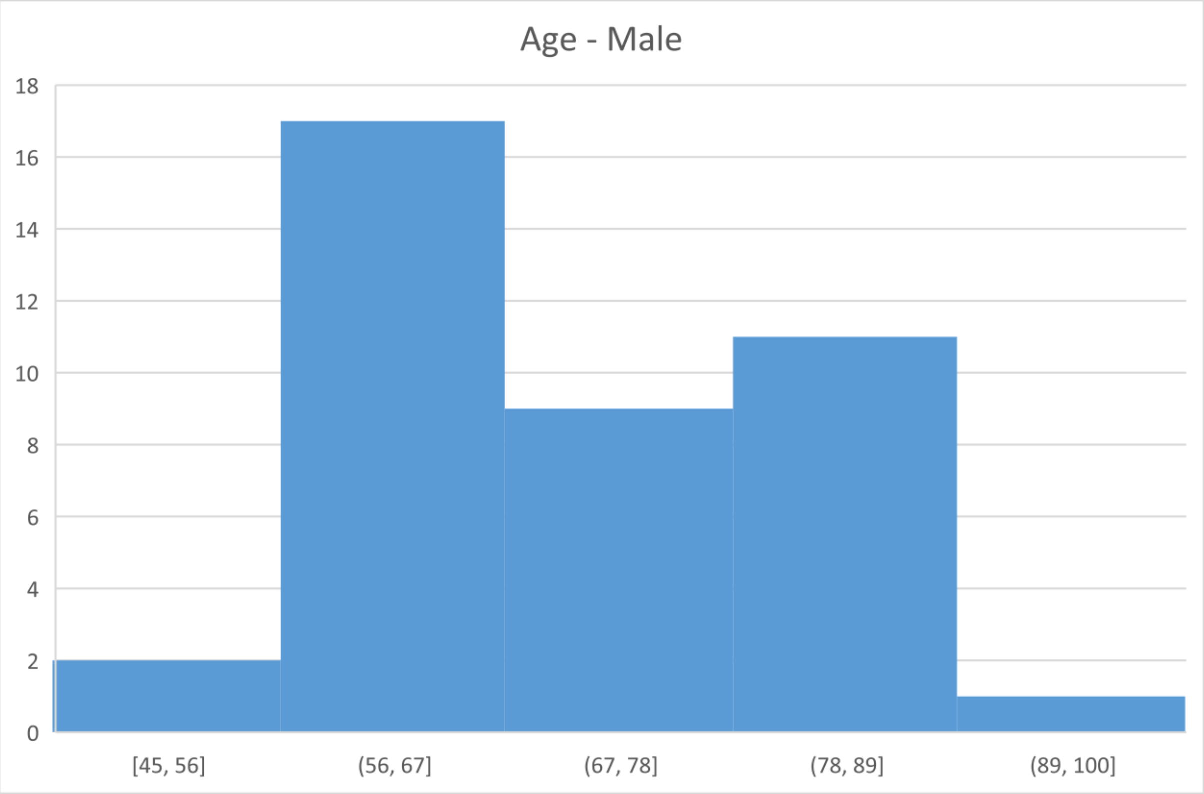

S1. Figure 3: Gender and age-based population data classification – Age Male.

IDs	Gender	Age
1073	Female	89
1084	Female	69
1087	Female	69
1088	Female	65
1093	Female	70
1097	Female	57
1105	Female	71
1108	Female	57
1111	Female	86
1114	Female	54
1116	Female	63
1058	Female	72
1064	Female	54
1065	Female	51
1069	Female	65
1071	Female	52
1072	Female	91
1074	Female	81
1075	Female	59
1078	Female	72
1082	Female	59
1059	Male	79
1068	Male	70
1085	Male	64
1086	Male	65
1089	Male	55
1090	Male	70
1091	Male	77
1092	Male	62
1094	Male	64
1095	Male	66
1096	Male	59

1098	Male	83
1099	Male	67
1100	Male	81
1101	Male	64
1102	Male	71
1103	Male	80
1104	Male	73
1106	Male	79
1107	Male	84
1109	Male	75
1110	Male	80
1112	Male	72
1113	Male	60
1115	Male	67
1117	Male	66
1118	Male	88
1060	Male	58
1061	Male	70
1062	Male	67
1063	Male	66
1066	Male	82
1067	Male	62
1070	Male	57
1076	Male	45
1077	Male	73
1079	Male	92
1080	Male	86
1081	Male	57
1083	Male	85

S1. Table 1: Gender and age-based population data classification.
