# Supplementary material for: RNA-seq driven expression and enrichment analysis to investigate CVD genes with associated phenotypes among high-risk heart failure patients
Source: Hum Genomics. 2021 Nov 13;15:67. doi: 10.1186/s40246-021-00367-8 (PMC8590246; doi:10.1186/s40246-021-00367-8)
Supplement: Supplementary file 2 — Additional file 2. High resolution figures. [file 40246_2021_367_MOESM2_ESM.pdf]

Supplementary material 2:

High resolution figures.

Title

RNA-seq driven expression and enrichment analysis to investigate CVD genes with associated phenotypes among high-risk Heart Failure patients.

Authors

Zeeshan Ahmed[1,2,4,5*], Saman Zeeshan[3], and Bruce T. Liang[5]

Affiliations

1. Rutgers Institute for Health, Health Care Policy and Aging Research, Rutgers University, 112 Paterson St, New Brunswick, NJ, USA.

2. Department of Medicine, Robert Wood Johnson Medical School, Rutgers Biomedical and Health Sciences, 125 Paterson St, New Brunswick, NJ, USA.

3. Rutgers Cancer Institute of New Jersey, Rutgers University, 195 Little Albany St, New Brunswick, NJ, USA.

4. Department of Genetics and Genome Sciences, UConn Health, 400 Farmington Ave, Farmington, CT, USA.

5. Pat and Jim Calhoun Cardiology Center, UConn School of Medicine, University of Connecticut Health Center, 263 Farmington Ave, Farmington, CT, USA.

[*]Corresponding author: Zeeshan Ahmed, Rutgers Institute for Health, Health Care Policy and Aging Research, Rutgers University, 112 Paterson Street, New Brunswick, 08901, NJ, USA. (zahmed@ifh.rutgers.edu and zahmed@uchc.edu).

Supplementary material 2: High resolution figures

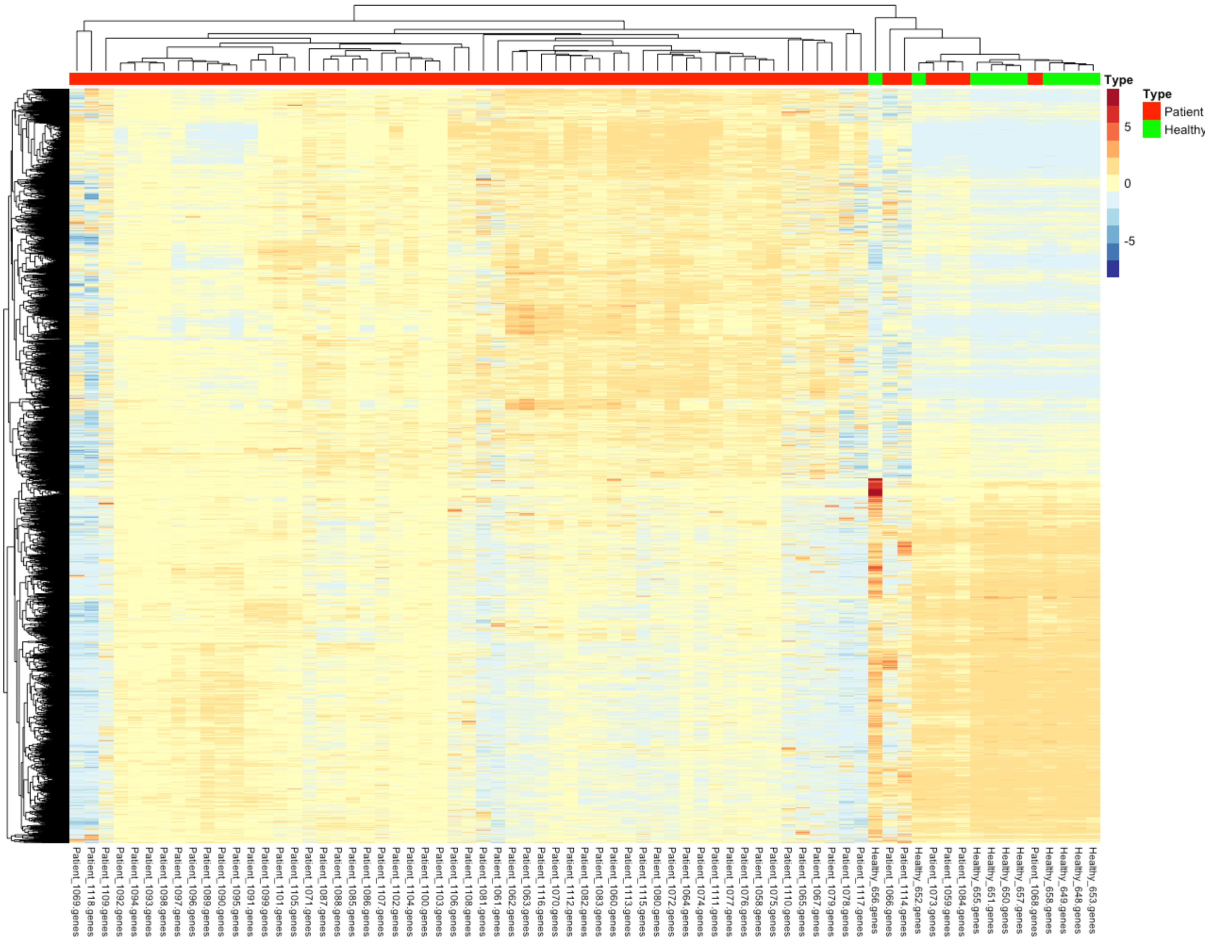

A: Differentially regulated gene expression analysis.

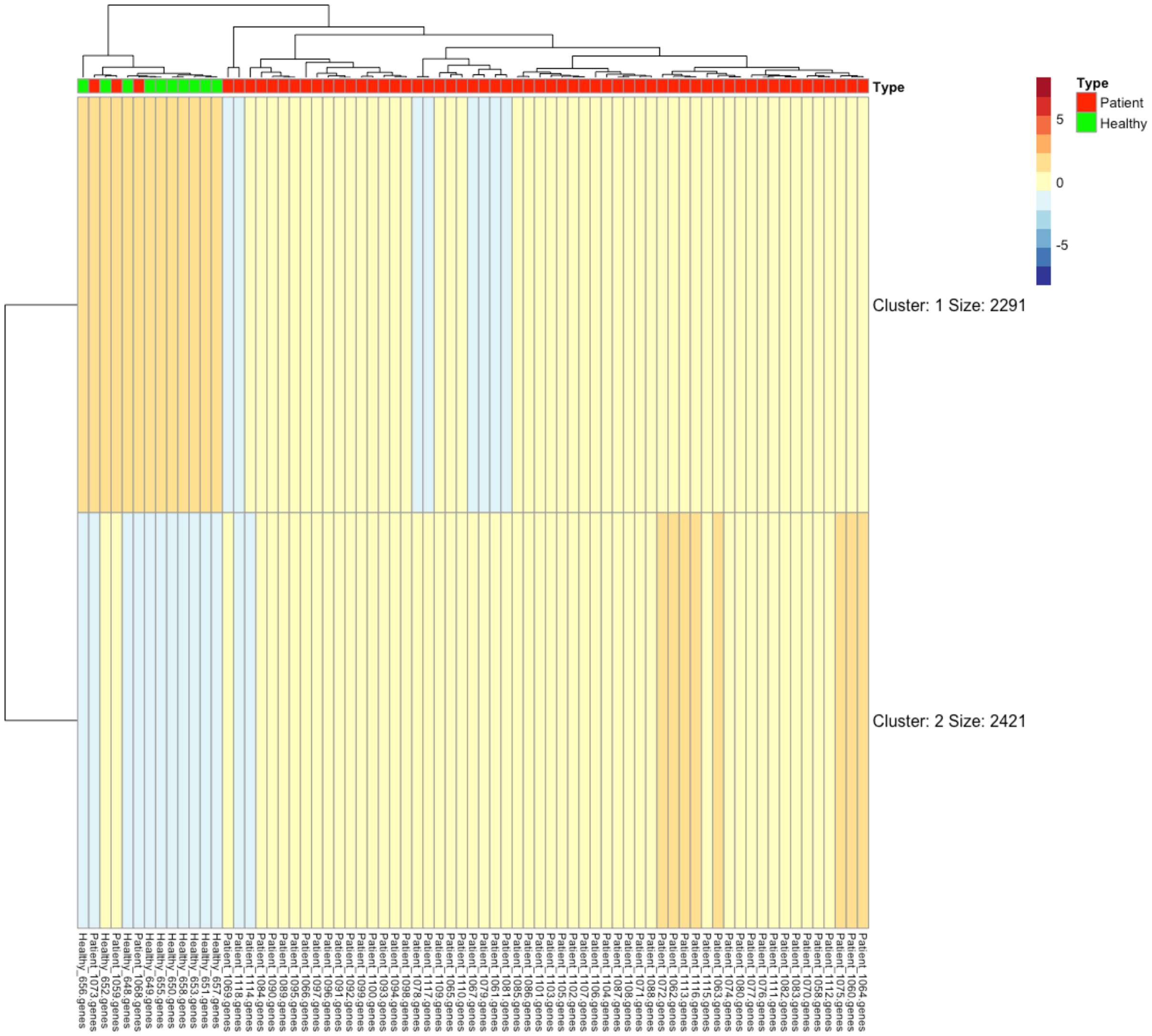

B: Differential gene expression of protein coding genes with two major clusters.

S. Figure 1. Differentially regulated gene expression analysis.

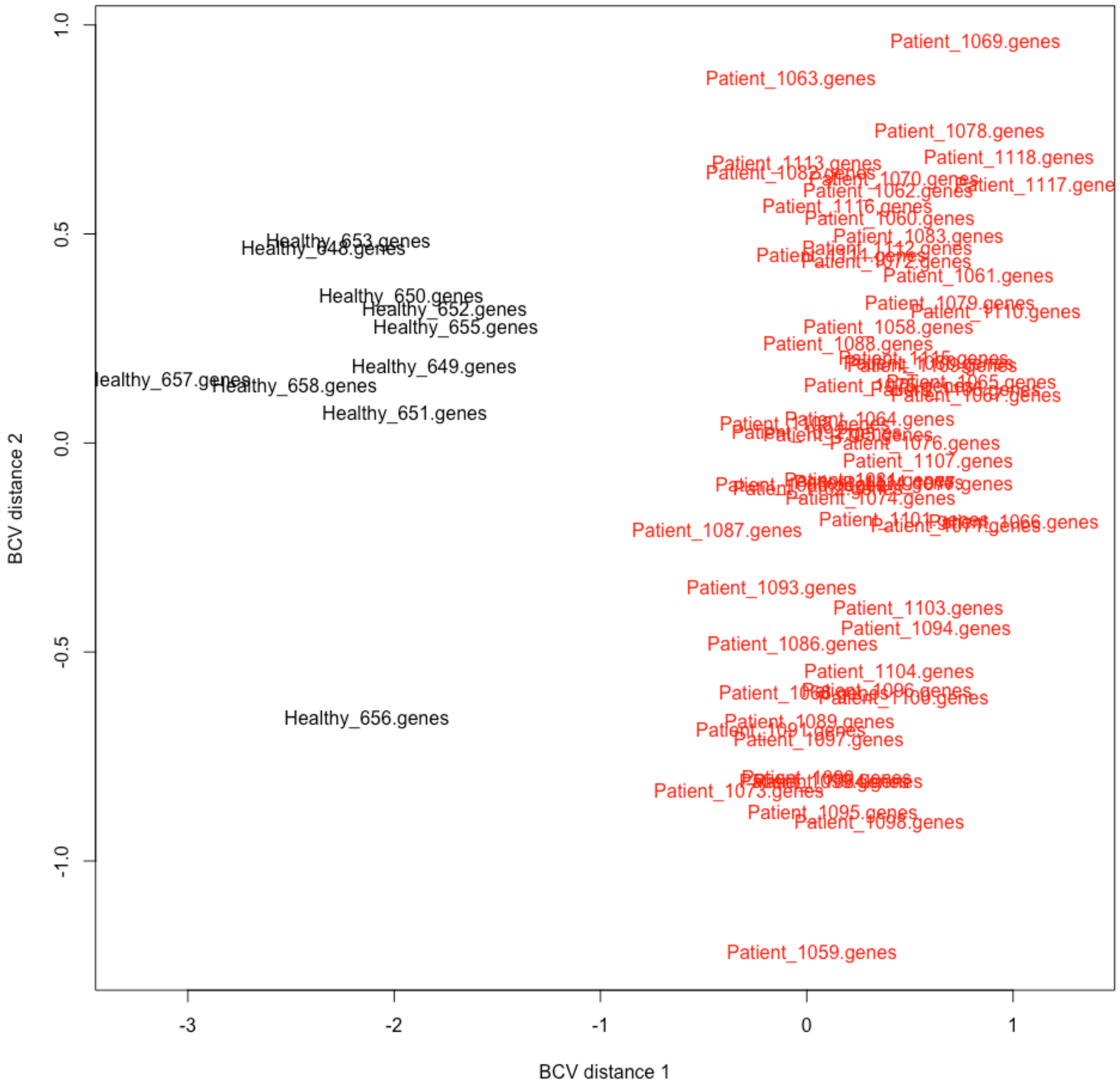

S. Figure 2. Multidimensional scaling (MDS) plotting of Biological coefficient of variation (BCV) distances.

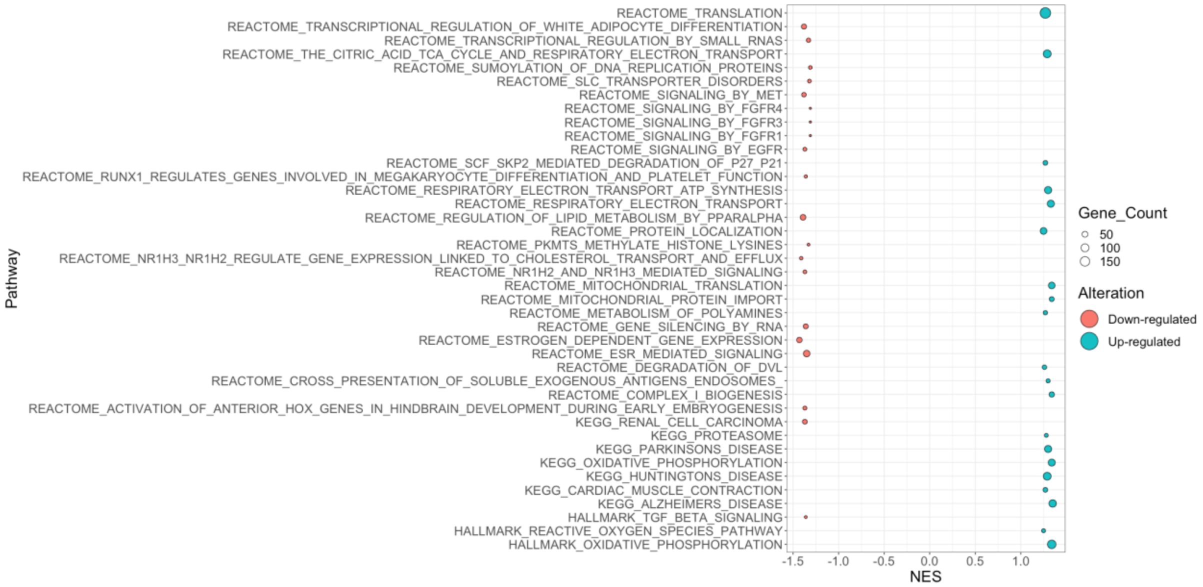

S. Figure 3. Upregulated and down-regulated pathways.

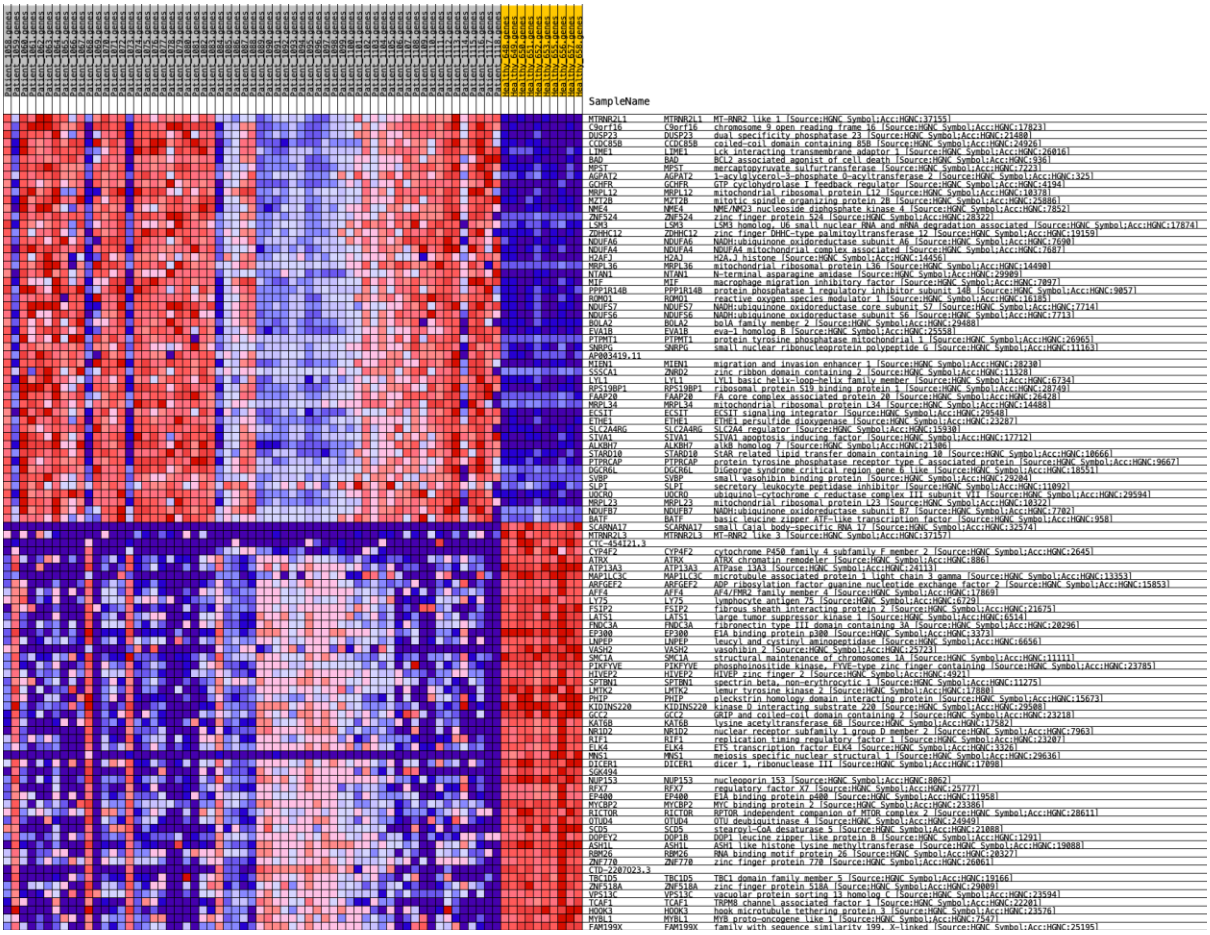

S. Figure 4. Annotated CVD genes.

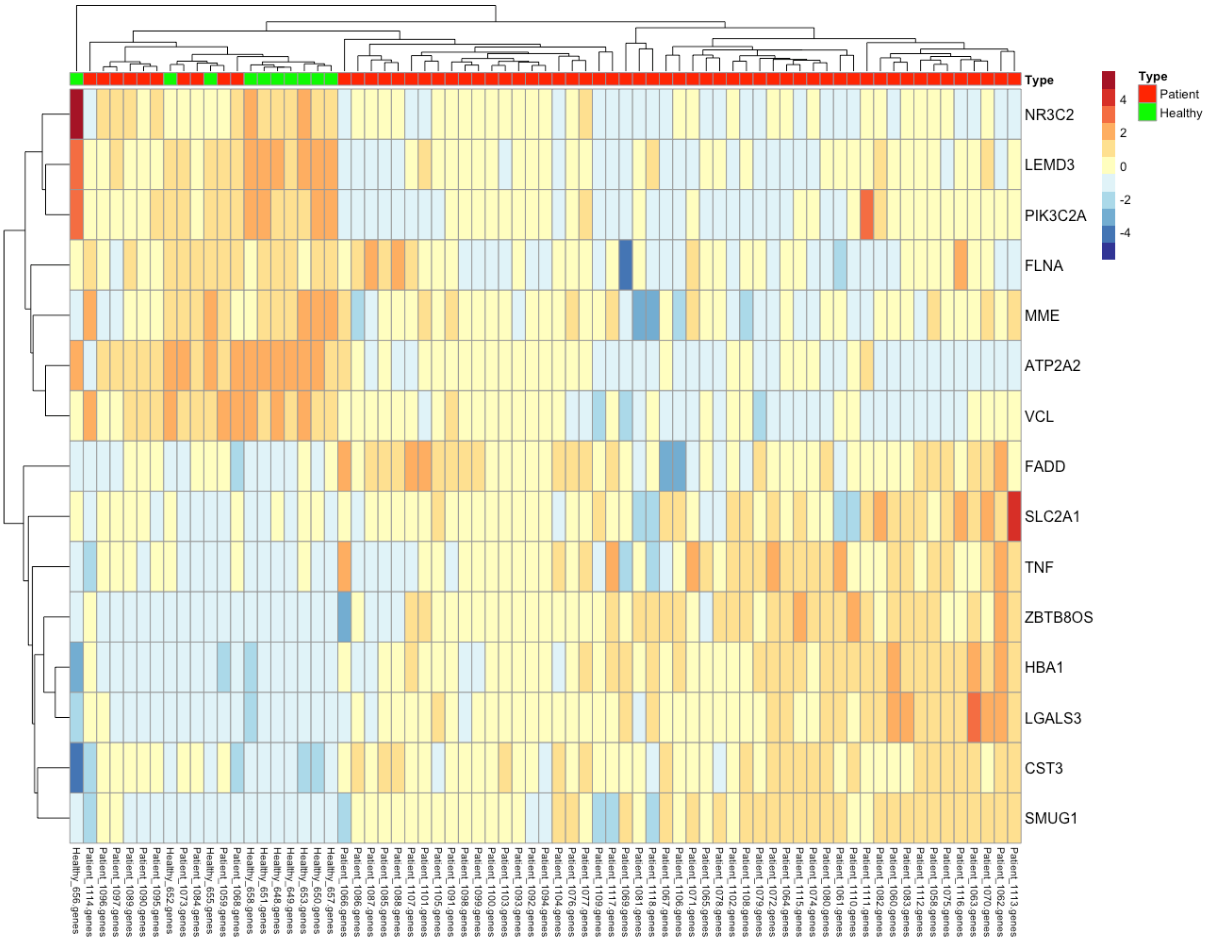

S. Figure 5. Gene enrichment analysis.

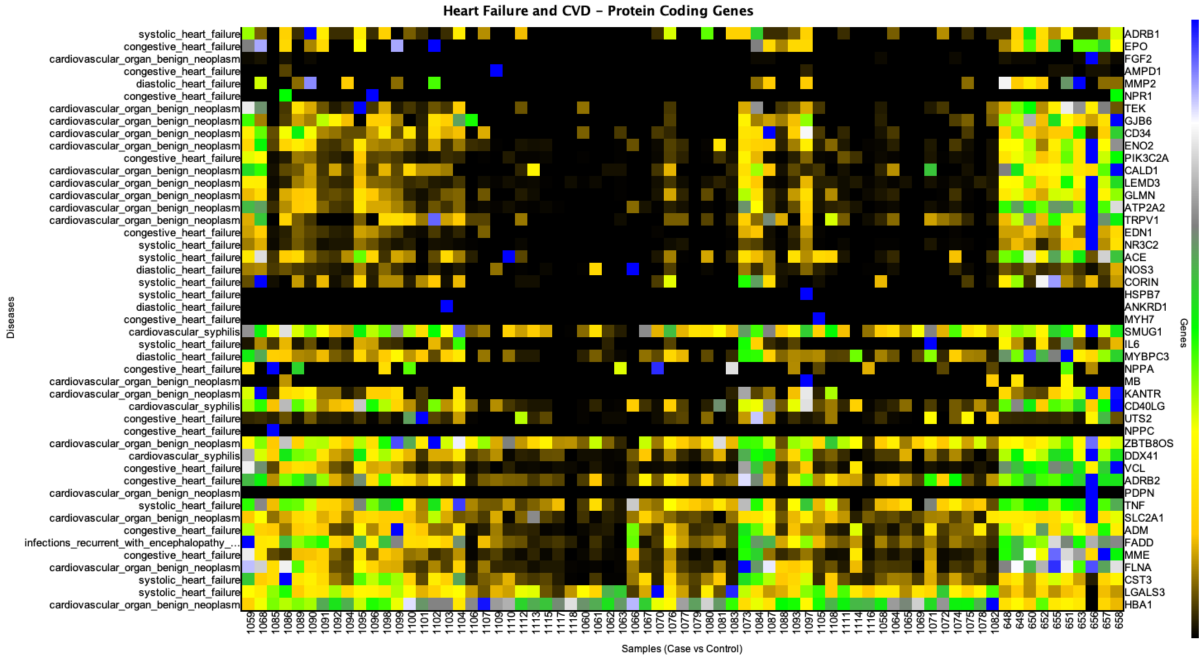

S. Figure 6. Genes-diseases heatmap for the expression analysis of CVDs among all diseased and healthy control patients.

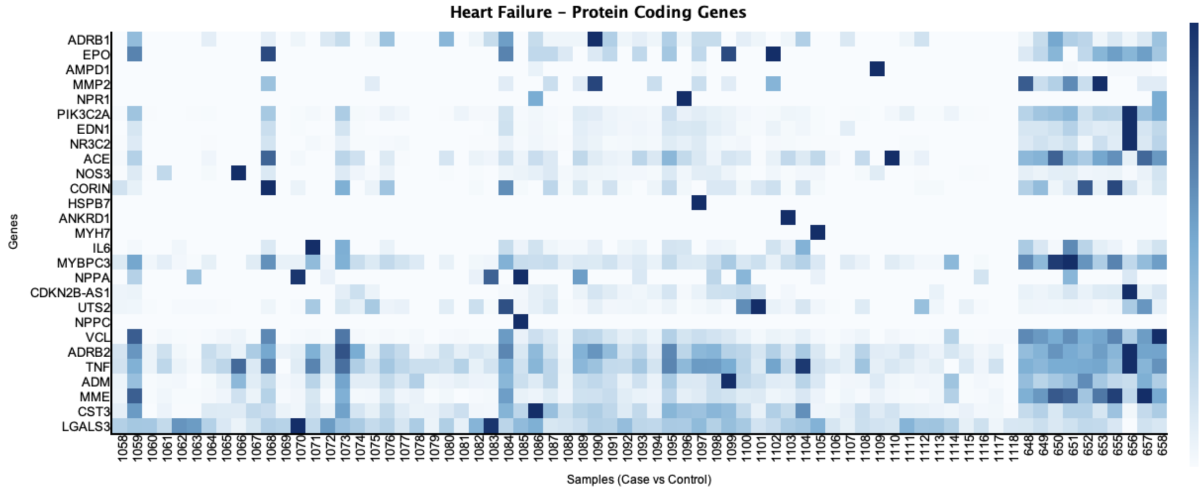

S. Figure 7. Unsupervised gene expression analysis of HF protein-coding genes.

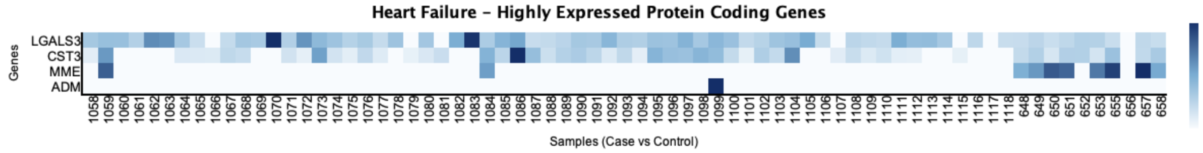

S. Figure 8. Unsupervised gene expression analysis of HF protein-coding highly expressed genes.

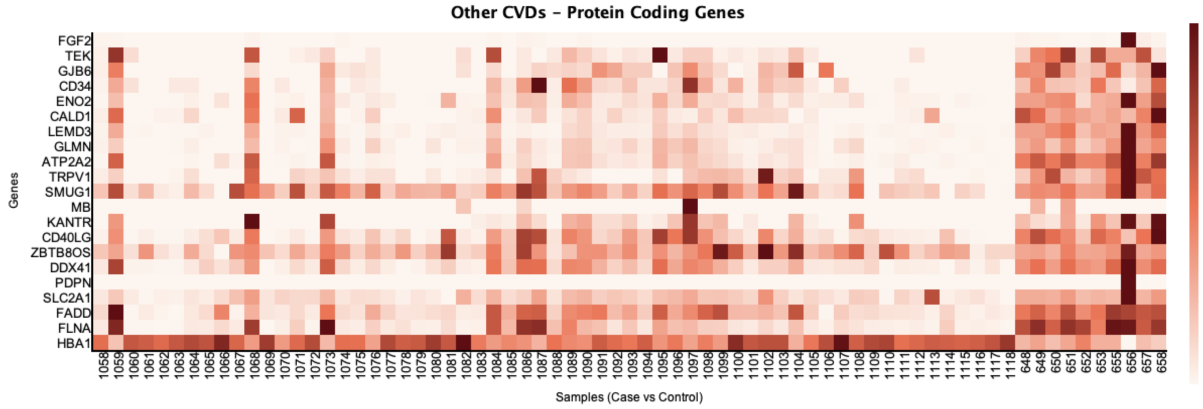

S. Figure 9. Unsupervised gene expression analysis of other CVD protein-coding genes.

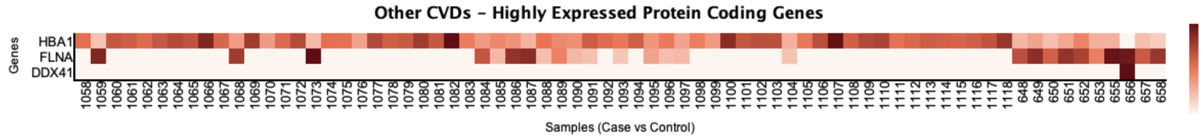

S. Figure 10. Unsupervised gene expression analysis of other CVD protein-coding highly expressed genes.

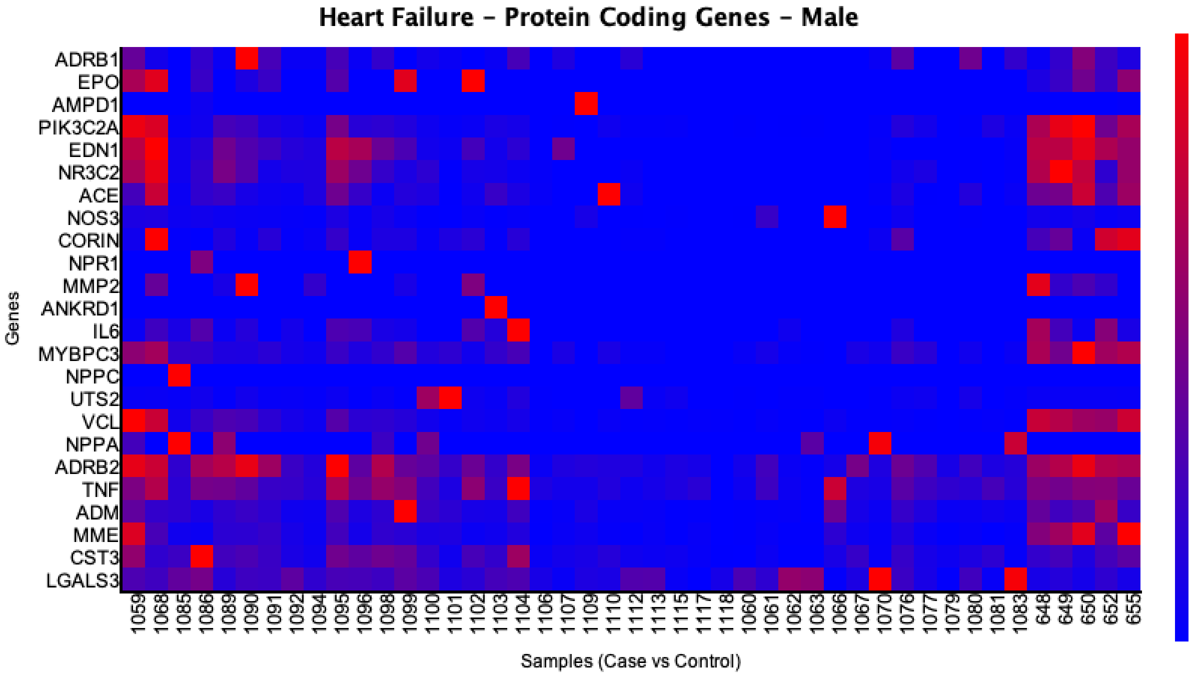

S. Figure 11. Protein-coding genes related to HF in males.

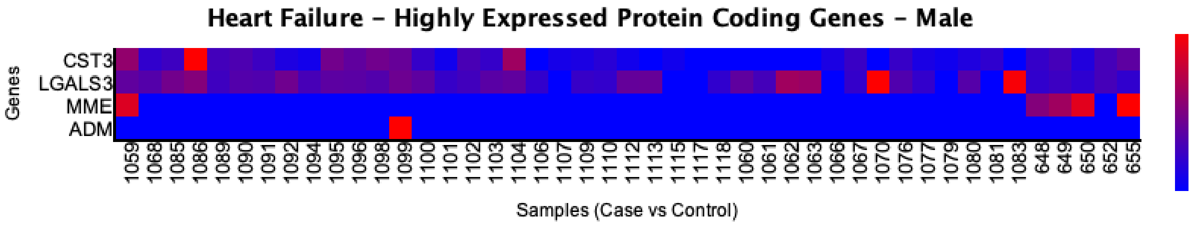

S. Figure 12. Highly expressed protein-coding genes related to HF in males.

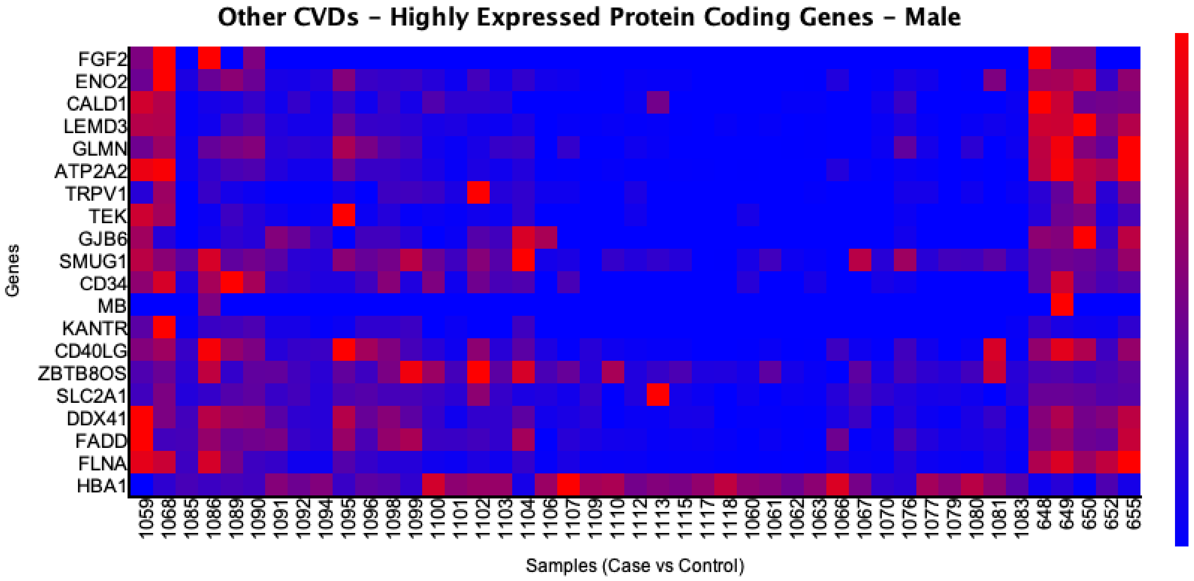

S. Figure 13. Protein-coding genes related to CVD in males.

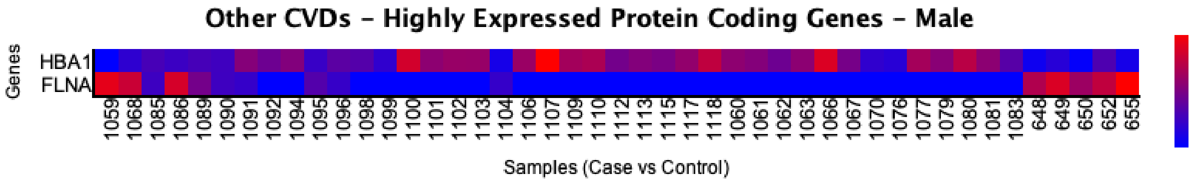

S. Figure 14. Highly expressed protein-coding genes related to CVD in males.

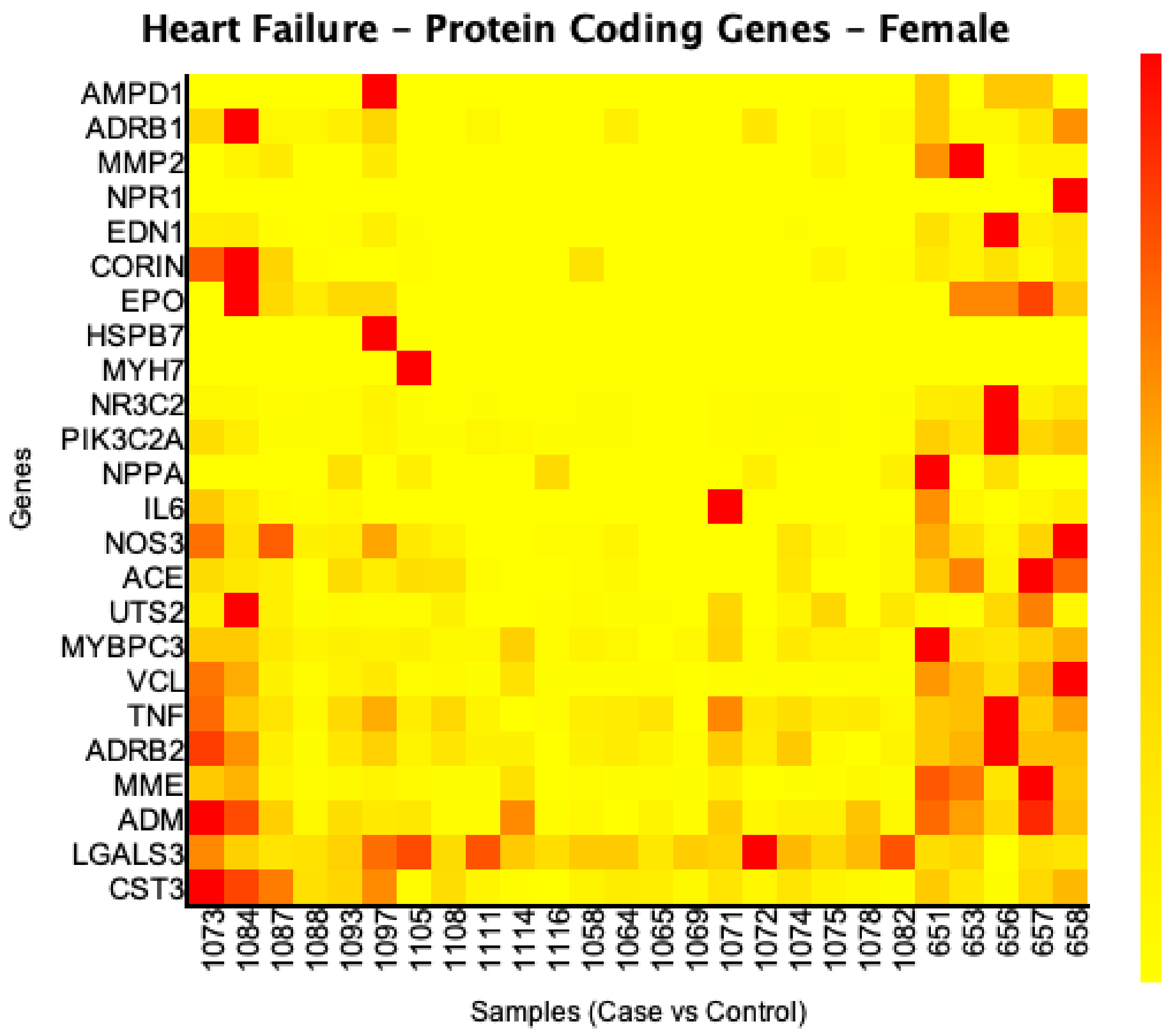

S. Figure 15. Protein-coding genes related to HF in females.

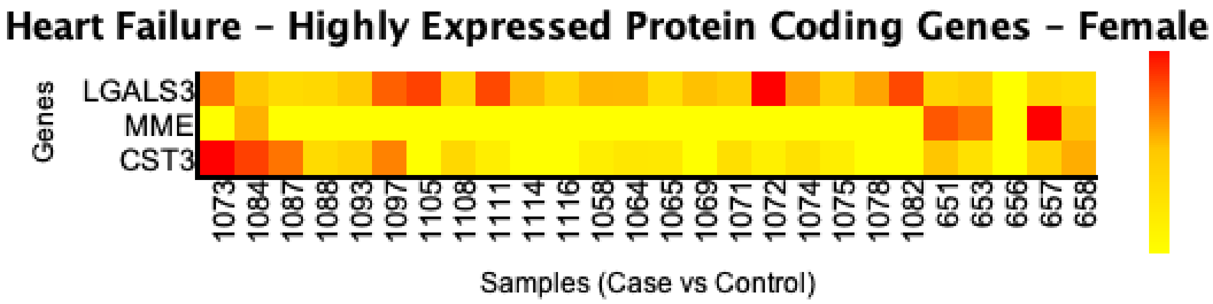

S. Figure 16. Highly expressed protein-coding genes related to HF in females.

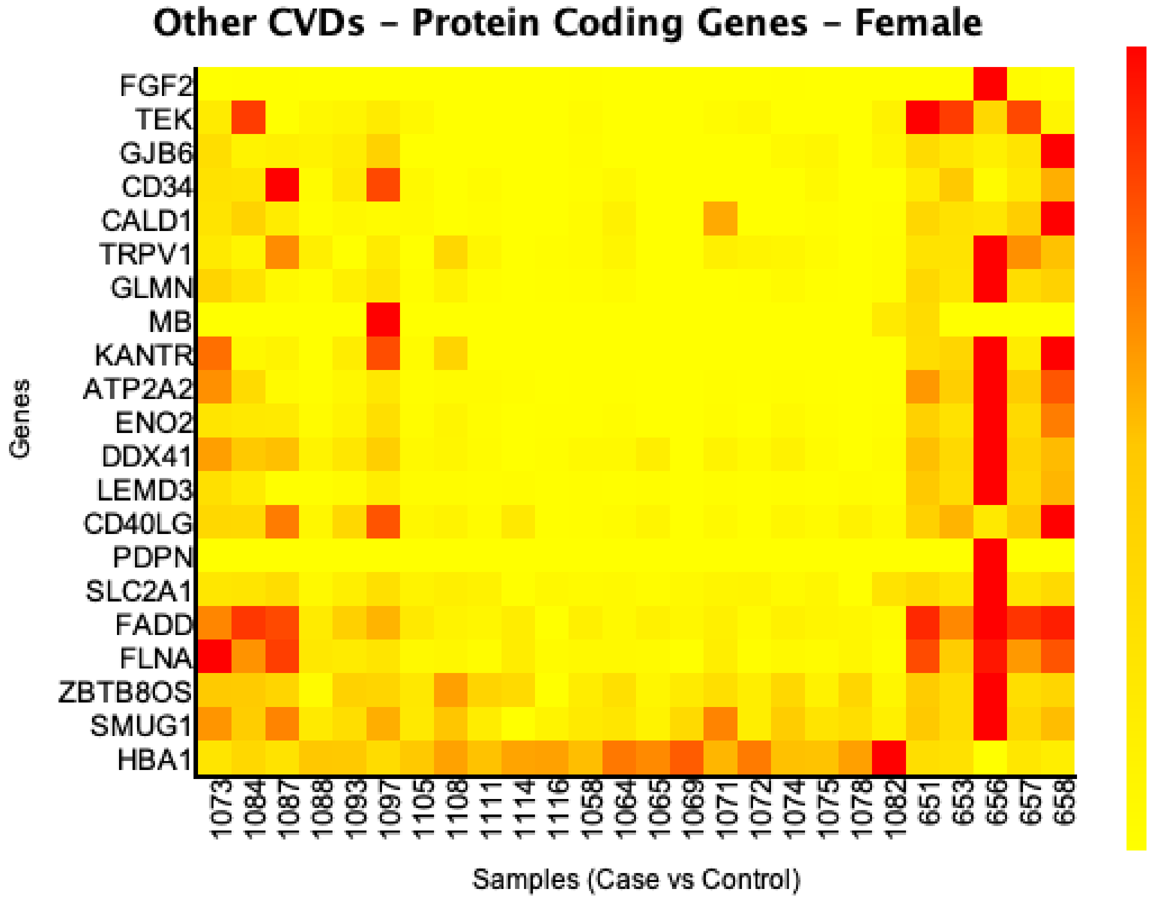

S. Figure 17. Protein-coding genes related to other CVD sin females.

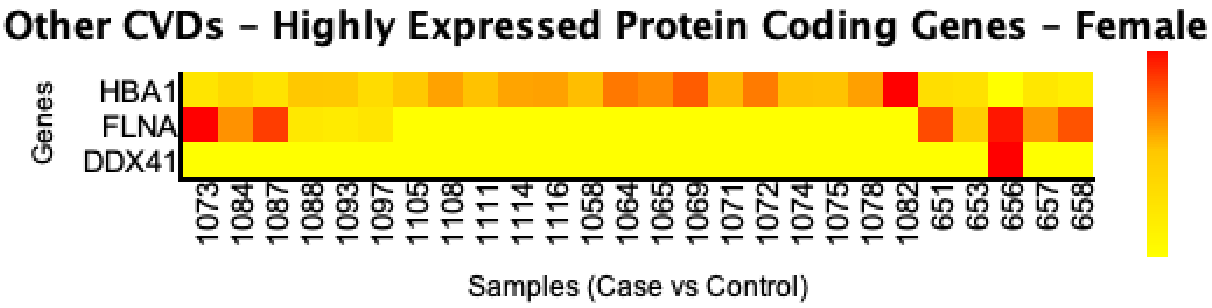

S. Figure 18. Highly expressed protein-coding genes related to other CVDs in females.

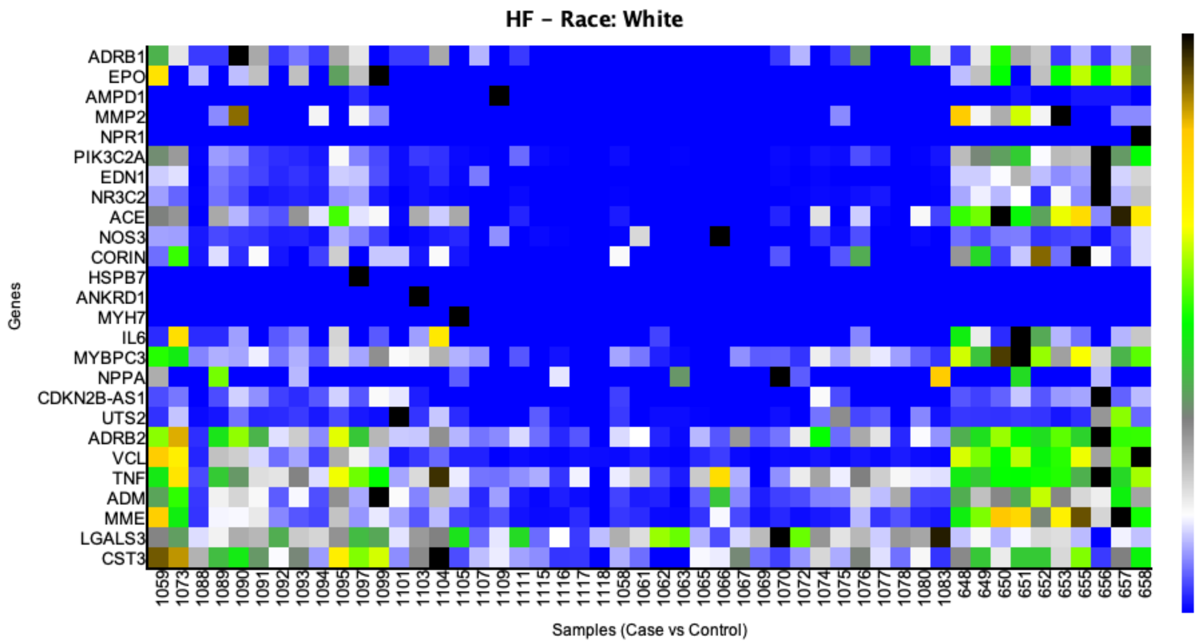

S. Figure 19. Expressed protein-coding genes related to HF in White American.

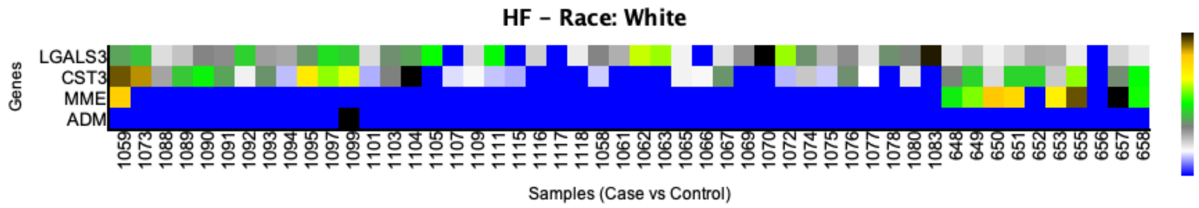

S. Figure 20. Highly expressed protein-coding genes related to HF in White American.

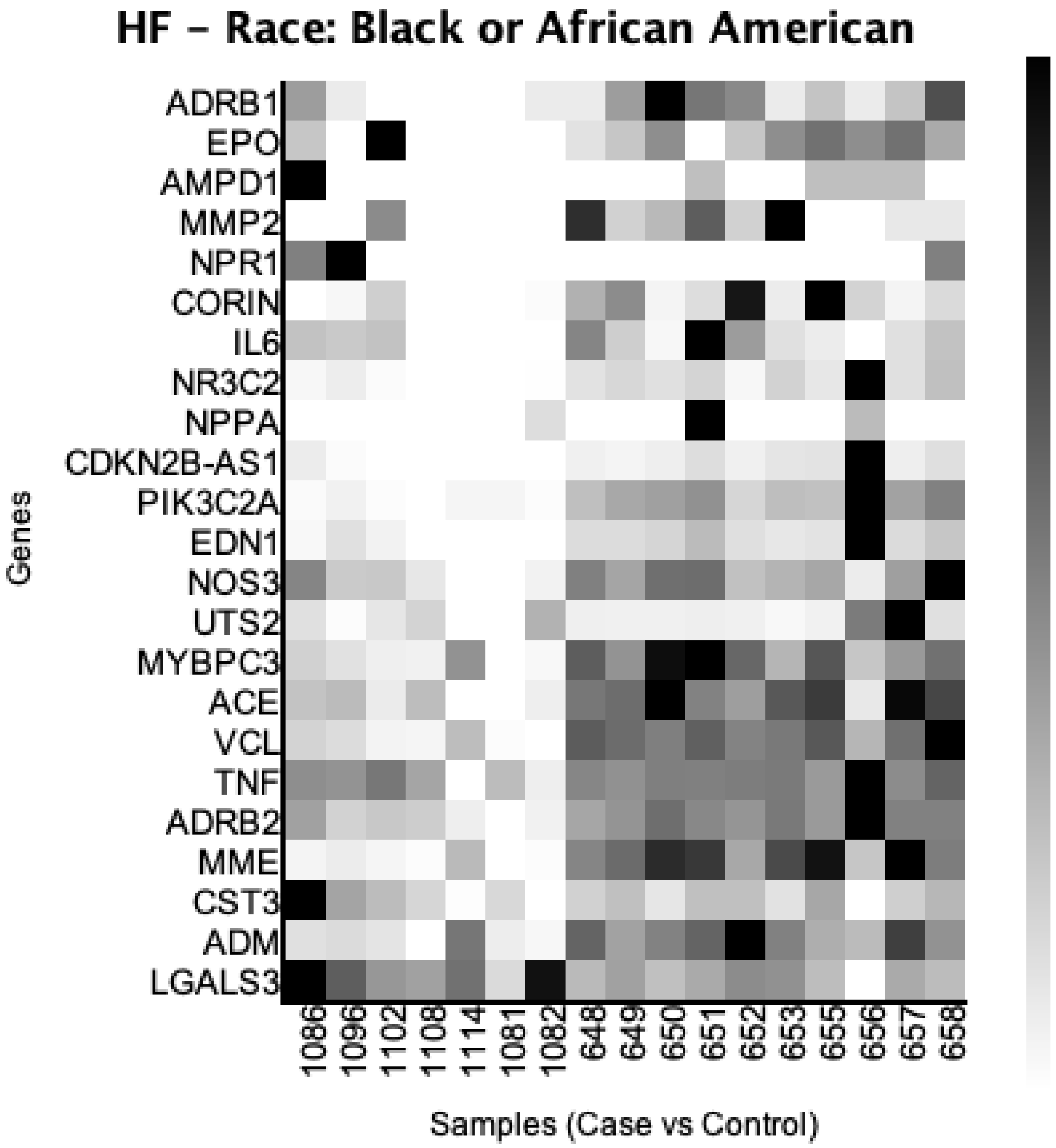

S. Figure 21. Expressed protein-coding genes related to HF in Black or African American.

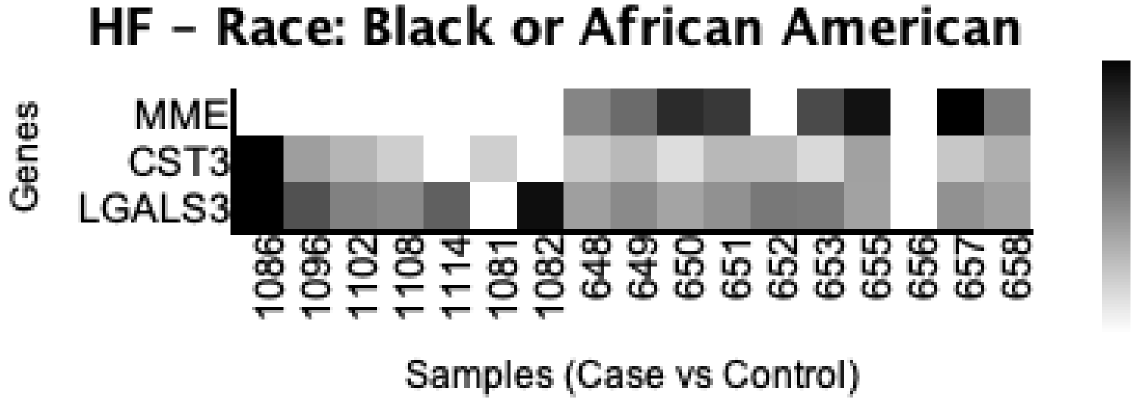

S. Figure 22. Highly expressed protein-coding genes related to HF in Black or African American.

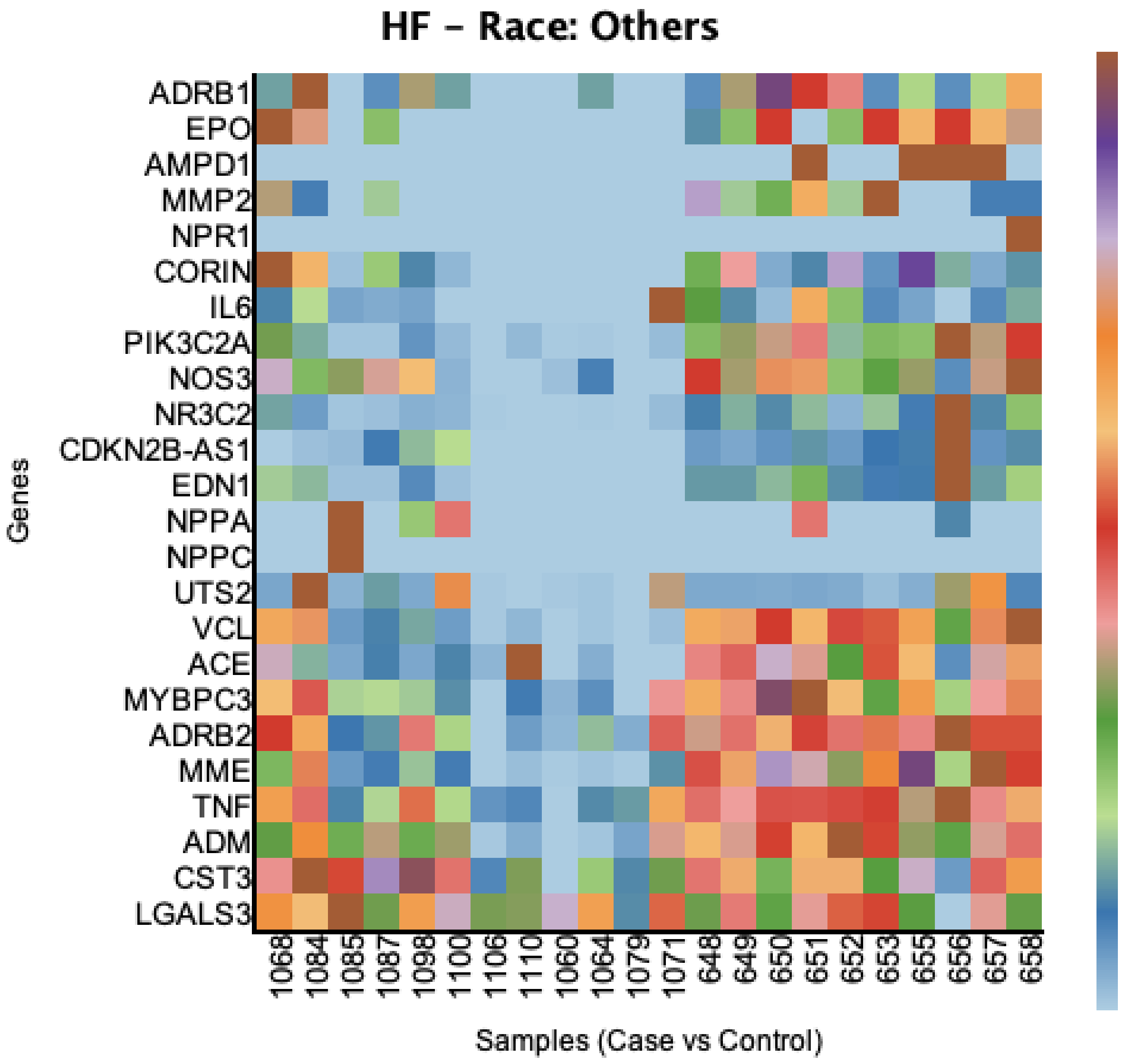

S. Figure 23. Expressed protein-coding genes related to HF in all other races.

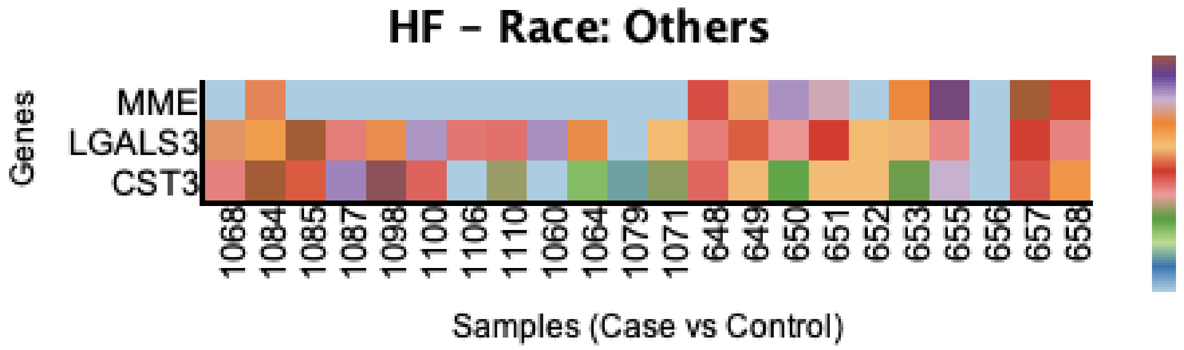

S. Figure 24. Highly expressed protein-coding genes related to HF in all other races.

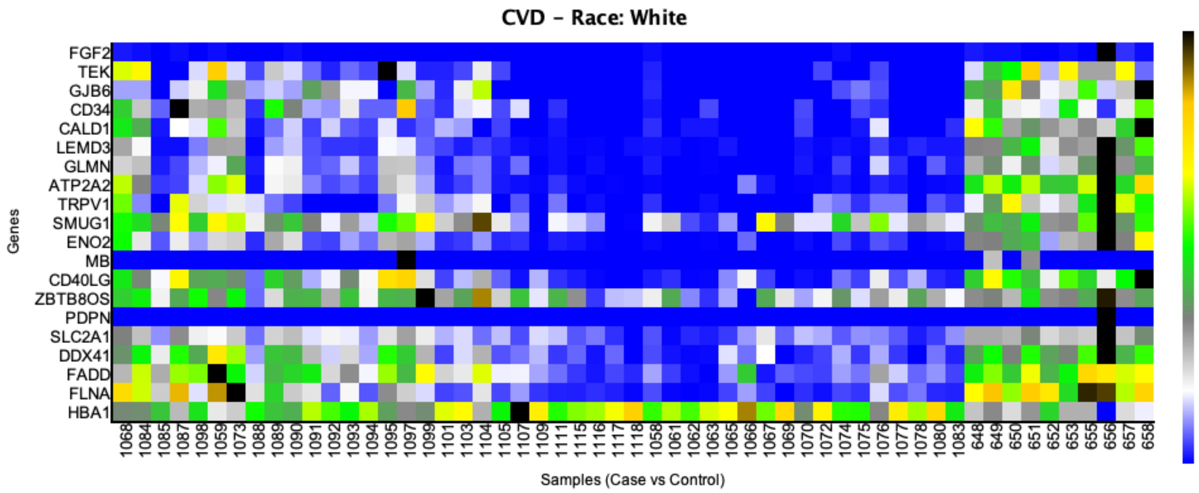

S. Figure 25. Expressed protein-coding genes related to other CVDs in White American.

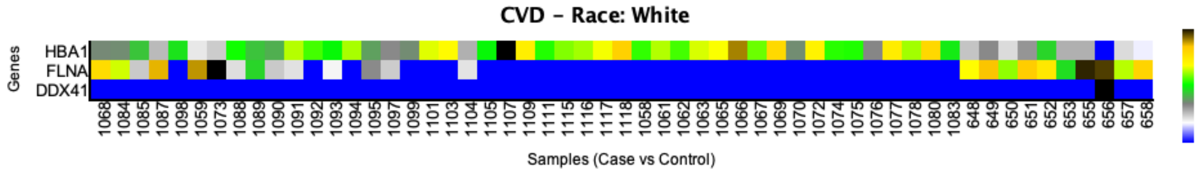

S. Figure 26. Highly expressed protein-coding genes related to other CVDs in White American.

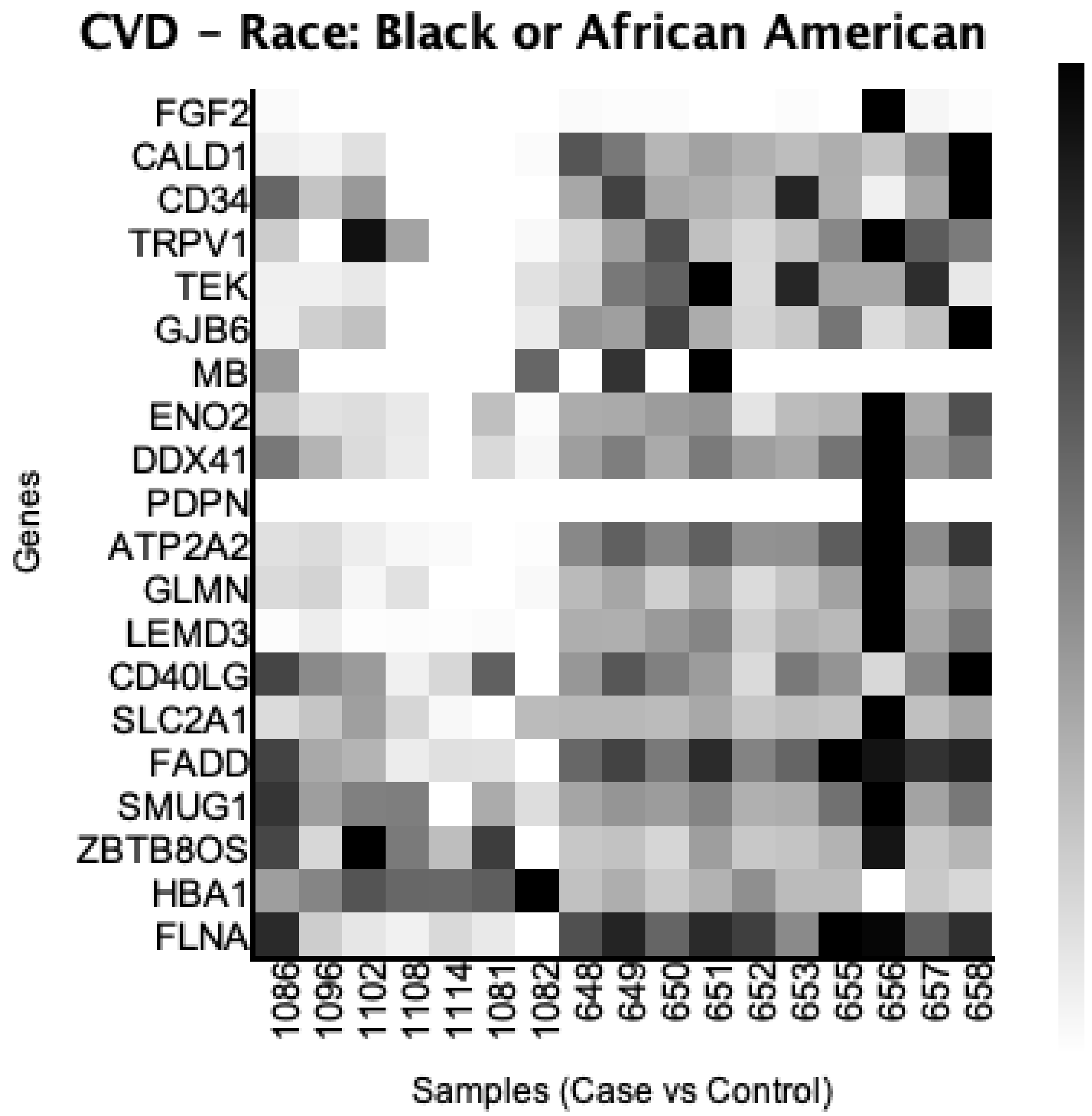

S. Figure 27. Expressed protein-coding genes related to other CVDs in Black or African American.

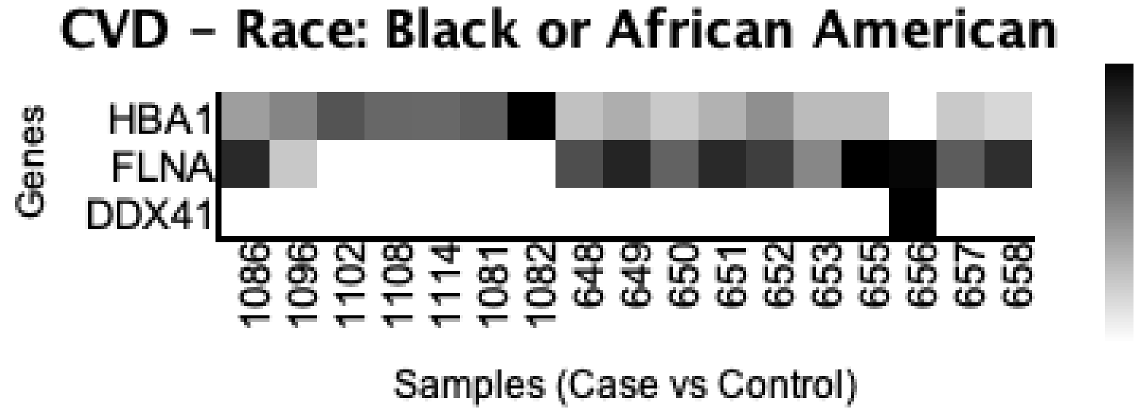

S. Figure 28. Highly expressed protein-coding genes related to other CVDs in Black or African American.

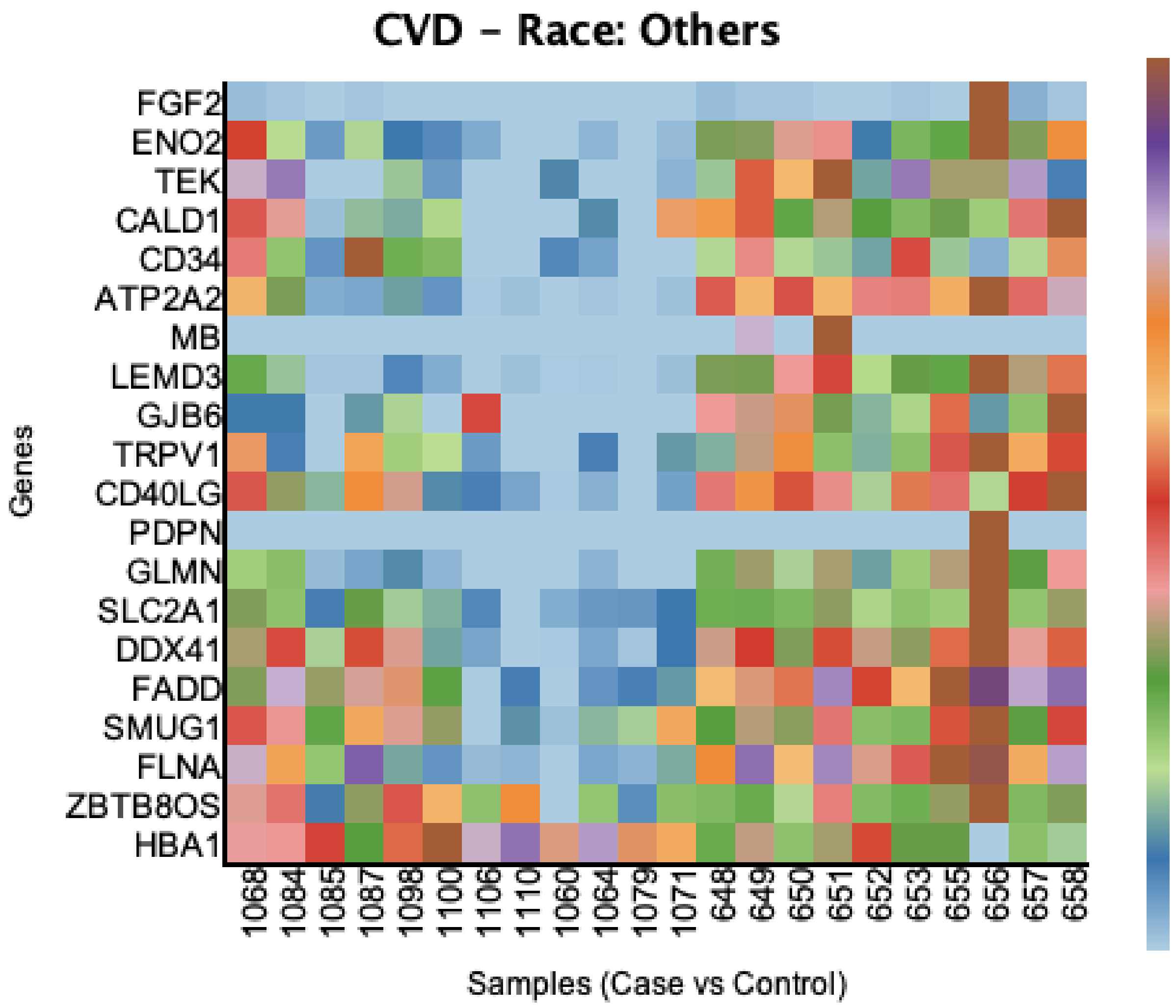

S. Figure 29. Expressed protein-coding genes related to other CVDs in all other races.

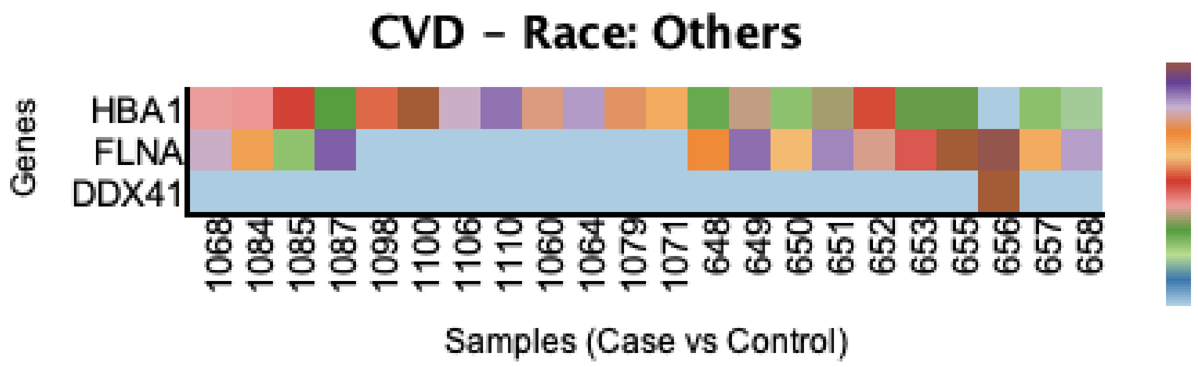

S. Figure 30. Highly expressed protein-coding genes related to other CVDs in all other races.
